# Supplementary material for: Community engagement in research addressing infectious diseases of poverty in sub-Saharan Africa: A qualitative systematic review
Source: PLOS Glob Public Health. 2024 Jul 15;4(7):e0003167. doi: 10.1371/journal.pgph.0003167 (PMC11249264; doi:10.1371/journal.pgph.0003167)
Supplement: S2 Checklist — (DOCX) [file pgph.0003167.s002.docx]

**S2 Checklist**: JBI Critical Appraisal Checklist of Qualitative Research
