# Supplementary material for: Community engagement in research addressing infectious diseases of poverty in sub-Saharan Africa: A qualitative systematic review
Source: PLOS Glob Public Health. 2024 Jul 15;4(7):e0003167. doi: 10.1371/journal.pgph.0003167 (PMC11249264; doi:10.1371/journal.pgph.0003167)
Supplement: S1 Table — (DOCX) [file pgph.0003167.s004.docx]

**S1 Table**: MEDLINE (Via PubMed) search strategy for community engagement in research (Last search date: July 10, 2023)

| Search | Query | Hits |
| --- | --- | --- |
| #1 | "Community Participation"[Mesh] OR “community participation”[tw] OR “community empowerment” [tw] OR “community based”[tw] OR “community engaged”[tw] OR “research community”[tiab] OR “community engagement”[tw] OR “citizen engagement”[tw] OR “citizen involvement”[tw] OR “stakeholder engagement”[tw] OR “stakeholders engagement” [tw] OR “stakeholders involvement” [tw] OR “stakeholders participation” [tw] OR "Stakeholder Participation"[Mesh] OR “community involvement”[tw] OR “community partnered”[tw] OR “community-led” [tw] OR “community led” [tw] OR “community action”[tw] OR “consumer participation”[tw] OR consumer-driven[tw] OR consumer driven[tw] OR “consumer involvement”[tw] OR “user involvement”[tw] OR “public participation”[tw] OR “patient engagement”[tw] OR “patient involvement”[tw] OR “participatory approach”[tw] OR “participatory method” [tw] OR “participatory tool” [tiab]OR “participatory techniques” [tiab] OR “community initiatives” [tiab] OR engage*[ti] OR community[ti] OR public[ti] OR "Social Participation"[Mesh] OR "Patient Participation"[Mesh] OR “patient empowerment” [tw] OR “clients engagement” [tw] OR “parents engagement” [tw] OR “families engagement” [tw] OR “community partnership” [tw] OR “academic-community partnership” [tiab] OR “community capacity development” [tiab] OR “community agent” [tiab] OR “community volunteer” [tiab] OR “community mobilization” [tiab] OR “capacity building” [tiab] OR “collaborative” [tiab] OR “co-creation” [tiab] OR “co-designs” [tiab] OR “co-learning” [tiab] OR “human-centered” [tiab] OR “human centered” [tiab] OR "Crowdsourcing"[Mesh] OR "Community Networks"[Mesh] OR “community advisory board” [tiab] OR "Rural Population"[Mesh] OR “community organizing”[tiab] OR “community engaged research” [tiab] OR "Social Planning"[Mesh] OR “community development” [tiab] OR "Research Subjects"[Mesh] OR "Research Personnel"[Mesh] OR "Minority Health"[Mesh] OR "Minority Groups"[Mesh] OR “community directed interventions” [tw] | 645,421 |
| #2 | "Translational Medical Research"[Mesh] OR "Biomedical Research"[Mesh] OR "Empirical Research"[Mesh] OR "Community-Based Participatory Research"[Mesh] OR “community-based participatory action research”[tw] OR “participatory action research”[tw] OR “Participatory research”[tw] OR “translational science”[tw] OR “translational medicine”[tw] OR “knowledge translation”[tw] OR “translational study”[tw] OR “translation of research”[tw] OR translational[ti] OR translating[ti] OR translated[ti] OR "Health Services Research"[Mesh] OR "Public Health Systems Research"[Mesh] OR "Qualitative Research"[Mesh] OR "Comparative Effectiveness Research"[Mesh] OR "Research"[Mesh] OR "Social Validity, Research"[Mesh] OR "Research Report"[Mesh] OR "Behavioral Research"[Mesh] OR "Ethics, Research"[Mesh] OR "Research Design"[Mesh] OR "Operations Research"[Mesh] OR "Interdisciplinary Research"[Mesh] OR "Citizen Science"[Mesh] OR "Nursing Research"[Mesh] OR "Pharmaceutical Research"[Mesh] OR "Dual Use Research"[Mesh] OR "Rehabilitation Research"[Mesh] OR "Patient Outcome Assessment"[Mesh] OR "Outcome Assessment, Health Care"[Mesh] OR “innovative research” [tw] OR “Implementation Research” [tw] OR “interventional study” [tw] | 2,438,009 |
| #3 | “poverty related diseases” [tw] OR “diseases of poverty” [tw] OR “tropical disease*” [tw] OR “tropical infectious disease*” [tw] OR “Infectious disease*” [tw] OR "Neglected Diseases"[Mesh] OR “Neglected Disease*” [tw] OR NTD [tw] OR helminthiasis [tiab] OR leprosy [tw] OR “lymphatic filariasis” [tw] OR onchocerciasis [tiab] OR trachoma [tw] OR [Trichomoniasis](https://en.wikipedia.org/wiki/Trichomoniasis) [tw] OR “[African trypanosomiasis](https://en.wikipedia.org/wiki/African_trypanosomiasis)” [tw] OR “[Chagas disease](https://en.wikipedia.org/wiki/Chagas_disease)” [tw] OR [Leishmaniasis](https://en.wikipedia.org/wiki/Leishmaniasis) [tw] OR [Dracunculiasis](https://en.wikipedia.org/wiki/Dracunculiasis) [tw] OR “[Guinea worm](https://en.wikipedia.org/wiki/Guinea_worm) disease” [tw] OR "Trichomonas Vaginitis"[Mesh] OR Leprosy [tw] OR Rabies [tw] OR “Buruli ulcer” [tw] OR Dengue [tw] OR Echinococcosis [tw] OR “Foodborne trematodiases” [tw] OR Taeniasis [tw] OR cysticercosis [tw] OR "Tuberculosis"[Mesh] OR "Acquired Immunodeficiency Syndrome"[Mesh] OR HIV/AIDS [tw] OR "Malaria"[Mesh] OR "Intestinal Diseases, Parasitic"[Mesh] OR "Schistosomiasis"[Mesh] | 748,239 |
| #4 | “Sub-Saharan Africa” [tw] OR Subsaharan [tw] OR Sub-saharan [tw] OR “Subsaharan Africa” [tw] OR "Africa South of the Sahara"[Mesh] | 273,452 |
| #5 | #1 AND #2 AND #3 AND #4 | 1,329 |
| #6 | *Filters applied: Humans, English, from 2005/1/1 - 2023/7/10* | 1,068 |
