# Supplementary material for: Community engagement in research addressing infectious diseases of poverty in sub-Saharan Africa: A qualitative systematic review
Source: PLOS Glob Public Health. 2024 Jul 15;4(7):e0003167. doi: 10.1371/journal.pgph.0003167 (PMC11249264; doi:10.1371/journal.pgph.0003167)
Supplement: S2 Table — (DOCX) [file pgph.0003167.s005.docx]

**S2 Table:** Characteristics of included studies

| **Study Reference** | **Study design, methods for data collection and analysis** | **Country** | **Phenomena of interest** | **Setting/context/culture** | **Participant characteristics and sample size** | **Description of main results** |
| --- | --- | --- | --- | --- | --- | --- |
| Agot et al. 2019 | (Content analysis)   - Engage study participants in dissemination sessions and one-on-one sharing sessions. A thematic approach was used and content analysis was performed. | Kenya and South Africa | TRIO participants’ views on the study results that assess the preferences for attributes of tablets, vaginal rings, and injectable products for dual prevention of HIV and pregnancy (TRIO Study) | Kisumu, Kenya and Soshanguve, South Africa | 277 TRIO study participants were invited to attend five dissemination sessions and five one-on-one sharing sessions | The dissemination sessions helped in giving context to the results and ensured correct lessons were derived from those results |
| Angwenyi et al. 2014 | (Qualitative approach to data collection and analysis, descriptive)   - Mixed methodology descriptive approach combining individual and group in-depth interviews and surveys, observations and document reviews - Interview data was analyzed thematically | Kenya | Experiences of formal CE for a pediatric randomized controlled malaria vaccine trial | Three sites in Kilifi County, Kenya | In-depth individual interviews with trial researchers (n = 5), community leaders (n = 8), parents (15 with enrolled children and 4 without); and group discussions with fieldworkers (n = 6) and facility staff (n = 2). Additionally, there was a survey of participating households (n = 200) and observed over 150 community engagement activities. | Majority perceived community engagement activities helped them to clear pre-existing concerns and misconceptions; increase visibility, awareness of, and trust in the trial staff. There was community engagement at different levels throughout the study period (i.e., consultation and sensitization with district stakeholders; community entry and sensitization with stakeholders in all sites; identification and recruitment of trial participants; follow-up of the research participants; continuous feedback to and from the community; and feedback on the results during the preliminary result dissemination). |
| Bandewar, Kimani and Lavery 2010 | (Qualitative approach to data collection and analysis, descriptive)   - Retrospective qualitative case study approach - Data was collected using in-depth interviews, non-participatory observation of meetings, and document review - Thematic and conceptual categories were as a method of analysis. | Kenya | Community engagement practices in the Majengo Observational Cohort Study (MOCS) that examined sexually transmitted infections, in particular HIV/AIDS, in a cohort of sex workers | Majengo, Kenya | 48 in-depth interviews with cohort members including peer leaders (n = 28), Majengo clinic staff (n = 6), researchers and project leaders (n = 6), research students (n=3), laboratory staff (n=2), research ethics board members (n=2), women’s group representatives and others (n=4). | The case study identified 3 distinct phases of community engagement in the Majengo Observational Cohort Study, MOCS: (1) reaching out: mobilization, dialogue, and education; (2) foundations of trust through relationships of care; and (3) leveraging existing social capital to form a cohort community. |
| Beard et al. 2020 | (Narrative)   - Narrative document from the experience of researchers, also drawing on project reports, public documents, and published articles | South Africa | Lessons from a community-engaged HIV/AIDS research project | Amajuba District, northern KwaZulu-Natal, South Africa | Researchers from two universities and community members in Amajuba District, northern KwaZulu-Natal, South Africa | Throughout the process, the project team was transparent about study goals and frequently communicated with community collaborators to understand local concerns about the research and engaged in ongoing review and modification of engagement strategies. |
| Broder et al. 2020 | (Qualitative approach to data collection and analysis, descriptive)   - The method of data collection is not clear, the paper describes the community engagement to aid recruitment in SSA (Botswana, Kenya, Malawi, Mozambique, South Africa, Tanzania, Zimbabwe) vs Americas/Switzerland (Brazil, Peru, Switzerland, and the United States) | Botswana, Kenya, Malawi, Mozambique, South Africa, Tanzania, and Zimbabwe | Strategies for community engagement to aid recruitment in HIV vaccine trials | Botswana, Kenya, Malawi, Mozambique, South Africa, Tanzania, and Zimbabwe | Community Working Group comprising 40 community representatives in Africa | Globally, referrals were the most efficient and effective recruitment strategy. Print materials were also valuable globally. In Africa, in-person outreach was quite effective and led to the most enrolments. Standardized metrics and data collection aid meaningful comparisons of optimal community engagement methods for trial enrolment. |
| Corneli et al. 2007 | (Qualitative approach to data collection and analysis, descriptive formative research)   - Formative research methods including semi-structured interviews, focus group discussions, home observations, and taste trials. Data was analyzed thematically. | Malawi | learn the attitudes and concerns of the local community on participating in research on the safety and efficacy of antiretroviral and nutrition interventions to reduce postnatal transmission of HIV | Lilongwe, Malawi | Semi-structured interviews were conducted with 40 HIV-positive mothers and with 35 mothers of undisclosed HIV status. All mothers had infants less than 1 year old. Additionally, 12 focus group discussions were conducted with 77 pregnant women, grandmothers, and fathers, and 33 semi-structured interviews were conducted with health providers, community leaders, and traditional birth attendants. | Participants were supportive of the clinical trial, although their overall understanding of the research was limited. The protocol was rapidly modified to achieve cultural acceptability while maintaining study objectives. |
| Davies et al. 2012 | (Qualitative approach to data collection and analysis, descriptive)   - Participatory method that has three phases: 1) review of existing forms, 2) form development with end-users, and 3) pretesting the form | Uganda | Engage target audience in problem-defining and solving process to design user-friendly adverse event reporting forms to capture information on events associated with artemisinin combination therapies (ACTs) for the treatment of malaria | Budondo a rural sub-county in Jinja and Kampala, Uganda | 10 community medicine distributors and 9 health workers | A novel adverse event form developed by engaging end-users during the problem-defining and solving process |
| Denison et al. 2017 | (Qualitative approach to data collection and analysis, descriptive)   - Brainstorming was the method for data collection. Secret voting was used to prioritize the research agenda | South Africa, Uganda, Zambia, South Africa, Tanzania, Mozambique | Youth engagement in developing an implementation science research agenda on adolescent HIV testing and care linkages | Sub-Saharan Africa | 4 youths living with HIV from southern Africa, 6 nongovernmental organizations, 7 researchers from US universities, and 6 international researchers from Africa | Youth participation influenced working group discussions and the development of the implementation science agenda. Research gaps identified included how to engage vulnerable adolescents, determining the role that stigma, peers, and self-testing have in shaping adolescent HTC behaviors, and examining the costs of different HTC and linkage to care strategies. |
| Diallo et al. 2005 | (Qualitative approach to data collection and analysis, descriptive)   - Describe the experience in the process used to obtain community permission | Mali | Obtaining community permission to conduct medical research (malaria vaccine study) | The malaria research site in Bandiagara, Mali, Africa | Bandiagara’s health authorities, the chief district administrator, the mayor, the neighborhood chiefs, traditional practitioners, school authorities, and religious leaders | The community permission-getting process had 6 steps: (1) a study of the community, (2) an introductory meeting with leaders, (3) formal meetings with leaders, (4) personal visits with leaders, (5) meetings with traditional health practitioners, and (6) recognition that obtaining permission is a dynamic process |
| Dierickx et al. 2018 | (Qualitative approach to data collection and analysis, descriptive)   - Data were collected using individual interviews, group discussions, and participant observation. - Interviews were systemized and analyzed with qualitative Analysis software. | Gambia | The relevance of community sensitization for individual decision-making in research participation in malaria transmission dynamics study | Rural Gambia | In-depth interviews (n=238) and informal conversations (n=17) were carried out with community members and field staff (1 policeman, 2 midwives, 2 blacksmiths, 3 Imam, 3 weavers, 3 griots (storytellers), 3 retired, 4 teachers, 4 treaders, 5 community health workers, 9 traditional birth attendants, 10 herders, 11 traditional healers, 13 Alkalo, and 198 farmers). Group discussions (n=9) were conducted when informants agreed to be interviewed together. | Community sensitization is effective in providing first-hand, reliable information to communities as the information is cascaded to those who could not attend the sessions. |
| Doshi et al. 2017 | (Qualitative approach to data collection and analysis, descriptive)   - In-depth interviews conducted through the snowball technique. - Transcripts analyzed thematically. | Kenya | The willingness of Kenyan men who have sex with men (MSM) to participate in HIV vaccine efficacy trials | Kisumu, Mombasa, and Nairobi | A total of 70 men who have sex with men (MSM) were interviewed from Kisumu (N = 20), Mombasa (N = 20), and Nairobi (N = 30). All were aged 18 years and above. | Most participants felt that an HIV vaccine would bring a number of benefits to themselves, as well as to MSM communities, including quelling personal fears related to HIV acquisition and reducing/eliminating stigma and discrimination shouldered by their community. Willingness to participate in HIV vaccine efficacy trials was highly motivated by various forms of altruism. |
| Faye and Lugand 2021 | (Qualitative approach to data collection and analysis, descriptive)   - Qualitative study combined IDI, FGD, and direct observation | Democratic Republic of Congo, Nigeria, and Mozambique | Participatory research for the development of information, education, and communication tools to promote intermittent preventive treatment of malaria | Kwango, DRC; Sofala, Mozambique; and Ebonyi, Nigeria | A total of 294 people were interviewed in all three countries, with 103 in-depth interviews and 191 through focus groups. Pregnant women, their husbands, as well as women who had recently given birth less than 6 months before; community health workers; and health authorities were part of the sample. | IEC tools were developed using an iterative and collaborative process, updating them by anticipating the user needs and making them user-friendly and adapted to social contexts that are useful to motivate healthcare providers to distribute the product at the community level. |
| Folayan et al. 2019 | (Qualitative approach to data collection and analysis, descriptive)   - Consensus-reaching process for the consultations using the Delphi method | Guinea, Liberia and Sierra Leone | Priorities for community engagement for research conducted during infectious disease outbreaks, Ebola | West Africa | A purposive sample of bioethicists, social scientists, researchers, policymakers, and laypersons who work with ethics committees in West Africa. | There is no presumptive justification for the exclusion of communities in the design, implementation, and monitoring of clinical trials conducted during an infectious disease outbreak. Engagement that facilitates collaboration rather than partnership between researchers and the community during epidemics is acceptable. |
| Freudenthal et al. 2006 | (Qualitative approach to data collection and analysis, descriptive)   - Participatory action research (PAR) with school activities that include school essay writing, video-recorded dramas, and household sanitation observations to prevent schistosomiasis | Tanzania | Participatory action research in primary schools in order to create enabling environments for the schoolchildren and other community members to adopt practices relevant to reducing the transmission of schistosomiasis | Mwanga district, Tanzania | School children (n=1146) in two primary schools in northern Tanzania | PAR is a mutual learning process. During repeated feedback meetings at the two schools and in the community, data has been shared, obstacles have been identified, and problems and solutions discussed among teachers, pupils, community members, and our research team. Teachers develop a curriculum for enhanced schistosomiasis education in primary schools, making household sanitation surveys part of school activities, better links between the school and the community, and actions taken by community members to create safe swimming places. |
| Hartley et al. 2021 | (Qualitative approach to data collection and analysis, descriptive)   - Qualitative analysis of interviews complemented by observation and documentary research | Mali | investigate how technology ‘co-development’ (between researchers, stakeholders, and local communities) is framed in practice by those developing gene drive mosquitos for malaria eradication | Mali | Researchers, stakeholders, and representatives of local communities | For Malians, co-development reflected Mali’s broader socio-political context and a desire for African scientific independence and leadership. It was mobilized to secure community and stakeholder support for gene drive mosquito field trials, through outreach, building local scientific capacity, and developing those institutions (e.g., regulatory) necessary for field trials to go ahead. |
| Hullur et al. 2016 | (Qualitative approach to data collection and analysis, descriptive)   - Qualitative approach using FGDs. Analysis was done using framework analysis. | South Africa | Community–based participatory research (CBPR) methods to identify community perspectives on HIV, violence, and health surveillance | Agincourt Heath and Socio-Demographic Surveillance Site, north-east South Africa | 15 FGDs were held in total in three villages. | The degree to which participatory principles were adopted in the study was limited. Given the time and resources available, communities participated in identifying and defining health problems only. |
| Kamanda et al. 2013 | (Qualitative approach to data collection and analysis, descriptive)   - A case study describing how to engage the community and adapt community-based participatory research using the CBPR framework | Kenya | How CBPR approaches and principles can be incorporated and adapted into study designs and methods of longitudinal studies in SSA using OSCAR project, HIV-related research | Uasin Gishu County of Western Kenya | A cohort of orphans and separated children from communities representing 300 households, 20 charitable children's institutions, and 7 community-based organizations | Community engagement and participation were integral in refining the study design and identifying research questions that were impacting the community. Through the participation of village Chiefs and elders, we were able to successfully identify eligible households and randomize the selection of participants. |
| Mabunda et al. 2016 | (Qualitative approach to data collection and analysis, descriptive)   - Workshop and focus group discussions were used to collect data. - Participatory analysis used to compare findings within and across all the participants | South Africa | Explore barriers and facilitators to belated care seeking and on-adherence to TB treatment behavior | Limpopo Province, Capricorn, Mopani, and Sekhukhune districts | A total of 161 participated in the study. The workshop was conducted with the planning group comprising 33 TB coordinators at different levels. Focus group discussions were conducted with professional nurses (37), DOT supporters (34), community members (30), and patients (27) aged 19-59 years. | The magnitude of the challenges justifies the adoption of a participatory approach involving various stakeholders to develop an intervention to meet the community's needs. |
| Marsh et al. 2011 | (Qualitative approach to data collection and analysis, descriptive)   - Authors document the experiences drawn from the health research using in-depth interviews and FGDs | Kenya | The role of the community in international collaborative biomedical research (community perceptions in the genomic epidemiological study and malaria vaccine trial) | Kilifi district, Kenya | Individual and small group interviews with the group of eight male field workers responsible for informed consent and sample and data collection, and 22 families participating in the research | Drawing on the experience of working with local residents in a rural setting in Kenya identified two normative roles played by communities in the setting. First, taking individual informed consent seriously involves understanding and addressing the influence of communities in which individuals’ lives are embedded. Second, individual participation can generate risks and benefits for communities as part of the wider implications of research. |
| Martínez et al. 2018 | (Grounded Theory)   - In-depth Interview and Focus Group Discussion - The transcripts were made and a feminist interpretation of grounded theory was used. | Liberia | Barriers and opportunities for pregnant women to participate in malaria research | Pregnant women attending antenatal care at the Saint Joseph’s Catholic Hospital (SJCH). | In-depth interviews and focus group discussions were held with hospital staff, traditional community representatives, and pregnant women. | According to the participants, useful strategies to motivate pregnant women to consent to participate in malaria research could be providing evidence-based education on malaria and research to the general population and encouraging the engagement of traditional leaders in research design and community mobilization. |
| Meiring et al. 2019 | (Qualitative approach to data collection and analysis, descriptive)   - Meetings with different stakes and lessons learned and community engagement activities recorded qualitatively. | Malawi | Community engagement activities before the initiation of the typhoid conjugate vaccine trial | Blantyre, Malawi | Different meetings such as community advisory group (CAG) meetings, community leaders’ meetings, school-based meetings, community health committee meetings, community representative meetings, and community meetings are held at different times. | Community engagement was successful for trail activities and enables community awareness and recruitment. |
| Molyneux et al. 2016 | (Qualitative approach to data collection and analysis, descriptive)   - Meetings with participants | Kenya | Public/community engagement in health research with MSM, HIV/AIDS-related research | Kilifi district, Kenya | 26 individuals and 11 institutions attended a one and half a day meeting. The majority of the invitees had an experience of CE in health research involving MSM either as a researcher or as community liaison personnel. | Underlying challenges and dilemmas linked to the stigma and discrimination of men who have sex with men in Africa raise special responsibilities for researchers. Community engagement is an important way of identifying responses to these challenges and responsibilities but it presents important ethical challenges. |
| Morin et al. 2008 | (Qualitative approach to data collection and analysis, descriptive)   - Data collection included a review of secondary data, including academic publications and site-specific progress reports; observations at the research sites; face-to-face interviews with CAB members, research staff, and other key informants; and focus groups with study participants. | Zimbwabwe | Evolution of Community Advisory Boards and community partnerships at international research sites conducting HIV prevention trials | Chitungwiza, a town in Zimbabwe | Interview with: Research staff(n=6), Community Advisory Board members(n=6), Informal interviews (n=5) and Focus group with study participants (n=10) | The relationship between research and community has been greatly facilitated through the work of the Community Liaison Department, where staff oversees the interface of research with the Community Advisory Board (CAB) and the community at large. CABs expanded their original function and became advocates for broader community interests beyond HIV prevention. The participation and input of community representatives, in response to critical incidents that occurred at the sites over the past five years, helped to solidify partnerships between researchers and communities. |
| Mtove et al. 2018 | (Qualitative approach to data collection and analysis, descriptive)   - Community engagement levels at different levels recorded qualitatively | Five countries in sub- Saharan Africa: Benin, Kenya, Malawi, Tanzania, and Uganda | Lessons learned in multiple level stakeholder engagements in malaria clinical trials | Different settings or sites in five sub-Sahara Africa countries | It depends on the level of engagement (international, national, and at community level) | Engagement with key stakeholders at international and national levels enabled the Sponsoring Entities to address challenges by aligning the study design with the requirements of health and regulatory agencies and to understand and address healthcare infrastructure needs prior to trial initiation. Local stakeholder engagement, including community members, study participants, and family enabled the investigators to address challenges by ensuring that study design and conduct were adapted to local considerations and ensuring accurate information about the study aims was shared with the public. |
| Nakalega et al. 2021 | (Qualitative approach to data collection and analysis, descriptive)   - Consultative meetings - Data presented as summary notes | Kenya | Community perspectives on ethical considerations for involving adolescent girls in the biomedical HIV prevention research | Kampala, Uganda | Two stakeholder meetings with 125 adolescent girls and young women (AGYW) aged 16-21 years, two stakeholder engagement sessions with 140 adult community representatives, and one meeting with 50 adolescents and their parents. | Stakeholder engagement with diverse community representatives prior to conducting adolescent HIV prevention research is critical to collectively shaping the research agenda, and successfully recruiting and retaining adolescents in HIV clinical trials. |
| Nakibinge et al. 2009 | (Qualitative approach to data collection and analysis, descriptive)   - Experiences of authors as investigators involved in HIV research project; review of project documents and peer-reviewed publications were conducted and analyzed qualitatively | Uganda | Experiences of authors as investigators (including opportunities and challenges) of engaging communities in HIV research project | Rural, South West Uganda | Principal investigators who involved in HIV research project | Judged by criteria of longevity, acceptance, and scientific output, community engagement in the HIV research project in rural Uganda has been successful. Key opportunities arise from the long-term joint commitment of the project and the community over nearly 20 years, and the potential to accommodate research beyond HIV. Challenges arise from participation fatigue, countered by innovations for the community and investment in capacity development for staff, and from the need to balance community development expectations and the project focus on HIV research. |
| Nyika et al. 2010 | (Qualitative approach to data collection and analysis, descriptive)   - Community meetings with different community members | Burkina Faso, Mali, Gabon, and Tanzania | Engaging diverse communities in malaria vaccine trails | Different settings in Burkina Faso, Mali, Gabon and Tanzania | Several meetings with community members (senior investigators, village chiefs, village elders, adult participants, etc.) | Community engagement enables the two-way sharing of accurate information and ideas between researchers and researched communities, which helps to create an environment conducive to smooth research activities with an enhanced sense of research ownership by the communities. |
| Ogunrin et al. 2021 | (Grounded Theory)   - The methodological design, adapted from grounded theory, used the constant comparative method of data analysis; while normative conclusions were made using the symbiotic empirical ethics approach | Nigeria | Explore the views of stakeholders on community participation and informed consent processes in genomic research, Malaria related research | Southwest Nigeria | Participants were purposively selected from community members attending the research facility (50 community members participated in focus groups, 2 community rulers, 2 opinion leaders, 2 community health workers, and 30 biomedical researchers) | Data analysis revealed five main themes important for successfully engaging communities in genomic research: effective communication, diversity of community gatekeeping, trust, cultural integration of research, and conservation of the research setting. From these themes, we have developed a four-stage model of community engagement that covers all stages of the research process; namely, the Community Approach, Intermediate phase, Collaboration, and Post‐research Cordiality model (CICP). |
| Okello et al. 2013 | (Qualitative approach to data collection and analysis, descriptive)   - Qualitative study using in-depth interviews and FDGs. The authors' experiences and observations of practice were also included. | Kenya | Experiences of consent, assent and community engagement in implementing a large school-based cluster randomized trial | Rural districts (Kwale and Msambweni), Kenya | 17 in-depth interviews with members of district health management team (DHMT) and district education offices (DEO), 22 focus group discussions (FDGs) with key stakeholders including parents, teachers, and community health workers. Additional 3 FDGs were conducted with field staff (health workers, educational assessors, and community mobilizers) directly involved in trial implementation to gather their views and experiences of the consenting and intervention process. | A range of stakeholders within and beyond schools play a key role in school-based health trials. Community entry and information dissemination strategies need careful planning from the outset, and ongoing consultation and feedback mechanisms established in order to identify and address concerns as they arise. |
| Olaseha and Sridhar 2005 | (Qualitative approach to data collection and analysis, descriptive)   - Participatory-action-research that empowers people through regular meetings and group discussions | Nigeria | Communities involved in the participatory-action research in controlling urinary schistosomiasis | Ibadan, South-western Nigeria | Regular meetings and group discussions that actively involve the affected communities | All actors involved benefitted from the learning experiences and skills, which the participatory action process offered. |
| Pare et al. 2021 | (Qualitative approach to data collection and analysis, descriptive)   - Qualitative data collection via in-depth interviews and FDGs. | Burkina Faso | Engagement activities relevant to field trials on non-gene drive genetically modified  mosquitoes as well as an assessment framework (Malaria) | Bobo-Diaolasso | A series of 35 in-depth interviews with leaders, women, men, young people, administrative authorities, and public servants. Fifteen FDGs with leaders, minority ethnic groups, and youth were also conducted. | The stakeholder engagement process, including the assessments, was crucial to building trust and empowerment with directly affected communities and other key stakeholders. They demonstrate the importance of responsibility and accountability mechanisms that can provide public confidence in how the project has been engaging communities and stakeholders with the appropriate respect for their autonomy and deliberation process. |
| Reddy et al. 2010 | (Content analysis)   - Qualitative design using in-depth and focus group interviews. Interviews were transcribed and analyzed using content analysis | South Africa | Functions and operations of Community Advisory Boards (CABs) in HIV/AIDS vaccine trials. | Gauteng, Western Cape, North-west KwaZulu-Natal, and Eastern Cape. | In-depth interviews with 18 key informants and 2 FGDs with 30 CAB members. The research participants were CAB members, principal investigators, research staff, community educators, recruiters, ethics committee members, and trial participants, and who were actively involved in at least one clinical trial from the South African Aids Vaccine Initiative (SAAVI). | CABs are seen primarily to serve and be accountable to the community on the one hand or to the trial site and the researchers on the other. Generally, this paper came with four themes named 1) Purpose (protect community interests and advance research goals) 2) membership and representation 3) power and authority, and 4) source of support and independence |
| Rennie et al. 2017 | (Qualitative approach to data collection and analysis, descriptive)   - FGD was used for data collection | Kenya | Solicit opinions about appropriate ways of conducting HIV research with adolescents | Three regions of Siaya County in Western Kenya: Asembo, Karemo, and Yimbo. The county has the third highest HIV prevalence rate in Kenya. | A total of 68 individuals participated in Round 1 and/or Round 2 of the FGDs. Of the 68 participants, 28 were Community Advisory Board (CAB) members and 40 were Youth Advisory Board (YAB) members. Of the 28 CAB members, 18 participated in a CAB specifically for parents, and 10 participated in a CAB specifically for professionals. All 10 of the participants in the CAB of professionals worked with adolescents, and the group was comprised of 3 teachers, 2 peer educators, 2 counselors or social workers, 1 health care provider, 1 community mobilization worker, and 1 other professional. Of the 40 YAB members, 20 were minors (younger than 18 years old). | Participants in all FGDs regarded participation in research as largely beneficial for individual study participants and their communities. Only a few participants in the FGDs suggested that research participation is not straightforwardly beneficial. |
| Reynolds et al. 2011 | (Grounded theory)   - Focus Group discussion and In-depth Interview was the method for data collection. - Analyzed using an iterative, line-by-line approach based on the principles of grounded theory | Tanzania | The experiences of people participating in a malaria clinical trial | Muheza, Tanzania | Eight Focus-group discussions have been held with HIV-positive and HIV-negative people who have participated in the trial, and with HIV-positive people who were screened but who did not participate. in-depth interviews with staff conducting the trial and delivering HIV care at the hospital. | A disconnect between the information given to trial participants in the recruitment and consent process and their understanding of the trial and its aims,2. Participants reported overwhelmingly positive experiences of participating in the trial, largely based on their access to numerous tests and free treatment, as well as reimbursement for transport and telephone costs. The findings from this study raise questions about how information is presented and used in the recruitment and consenting process for a trial, particularly one located within an existing clinical context. Recruitment strategies should take into consideration when, where, and how the information will be conveyed to participants, and explore likely expectations of these contexts which may shape how and if the information is interpreted and utilized by potential participants. |
| Shahmanesh et al. 2021 | (Qualitative approach to data collection and analysis, descriptive)   - Data was collected qualitatively and analyzed thematically | South Africa | Community-Based Participatory Research to iteratively co-create and contextually adapt a biosocial peer-led intervention to support HIV prevention | Mkhanyakude district of KwaZulu- Natal (KZN) | Men and women aged 18–30 years who participated in the participatory intervention development workshops | Local youth were able to use evidence to develop a contextually adapted peer-led intervention to deliver biosocial HIV prevention |
| Silumbwe, Halwindi and Zulu 2019 | (Qualitative approach to data collection and analysis, descriptive   - An exploratory qualitative case study using FGD and in-depth interviews employed. A thematic approach was applied to analysis | Zambia | Explored how community engagement approaches used in Mass Drug Administration (MDA) for Lymphatic Filariasis (LF) shape participation in the program | Luangwa, a rural District of Lusaka Province, Zambia | A total of nine focus group discussions, six in-depth and seven key informant interviews were conducted with various participants that included; community members, traditional leaders, and program managers, respectively. Data were analyzed using a thematic approach, aided by NVivo 10 software. | Facilitating participation in MDA for LF will require designing and implementing effective community engagement strategies that take into account local context, but also seek to explore all avenues of maximizing participation for improved coverage levels. |
| Simwinga et al. 2016 | (Qualitative approach to data collection and analysis, descriptive   - Qualitative approach to describe the community engagement and lessons learned | Zambia and South Africa | Experiences of engaging communities in the design and implementation of a community-randomized study of combination HIV prevention | communities throughout Zambia and Western Cape, South Africa | Different consultative meetings and dialogues with civil society | Engaging the community from the very early stages has been critical to the acceptance of this large, community-randomized study. Early lessons from the HPTN 071 study demonstrate that CE is integral to facilitating communication, nurturing trust and dispelling myths and rumours; all of which are essential to the implementation and success of a community randomized study. |
| Tarr-Attia et al. 2018 | (Grounded Theory)   - In‑depth interviews and focus group discussions were conducted using a feminist interpretation of grounded theory | Liberia | The community and health workers’ perceptions on the utility of malaria research for pregnant women | Pregnant women attending first antenatal care at the Saint Joseph’s Catholic Hospital (SJCH). | Pregnant women, traditional community representatives, and hospital staff (n = 38). Seventeen pregnant women, 11 traditional community representatives, and 10 hospital staff participated in 26 IDIs and in three FGDs | The participants suggested that malaria research in Liberia could help to design evidence-based education to change current malaria prevention, diagnostic and treatment-seeking attitudes, and to develop more acceptable prevention technologies. |
| Vreeman et al. 2012 | (Qualitative approach to data collection and analysis, descriptive)   - Data were collected using group discussions (mabaraza) and data were analyzed manually | Kenya | Evaluating community understanding of research and processes like informed consent in a particular setting, HIV/AIDS-related research | Uasin Gishu county of western Kenya, It is located in the Rift Valley province. | The 108 mabaraza participants consisted of the provincial administration (the Chief, Assistant Chiefs, the District Children’s Officer, and village elders), caregivers of orphaned and separated children, and members of the general public, both male and female, including elderly caregivers. | Participants understood some principles of biomedical research; they emphasized perceived benefits from participation in research over potential risks. Many community members equated health research with HIV testing or care, which may be explained in part by the setting of this particular study. In addition to valuing informed consent as understanding and accepting a role in research activities, participants endorsed an increased role for the community in making decisions about research participation, especially in the case of children, through a process of community consent. |
| Yotebieng et al. 2019 | (Qualitative approach to data collection and analysis, descriptive)   - Delphi approach was applied to prioritize the research agenda | Sub-Saharan Africa | Develop consensus around research priorities for Treat All implementation in SSA, HIV/AIDS-related research | Sub-Saharan Africa | More than 200 researchers, implementation experts, policy/decision-makers, and HIV community representatives in East, Central, Southern, and West Africa. | Reflecting consensus among a broad group of experts, researchers, policy- and decision-makers, PLWH, and other stakeholders, the resulting research priorities highlight important evidence gaps that are relevant for ministries of health, funders, normative bodies, and research networks. |
