# Supplementary material for: Community engagement in research addressing infectious diseases of poverty in sub-Saharan Africa: A qualitative systematic review
Source: PLOS Glob Public Health. 2024 Jul 15;4(7):e0003167. doi: 10.1371/journal.pgph.0003167 (PMC11249264; doi:10.1371/journal.pgph.0003167)
Supplement: S3 Table — (DOCX) [file pgph.0003167.s006.docx]

**S3 Table**: Summary of findings on community engagement activities/experiences in research

| **Reference and Country study conducted** | **Research focus** | **Community engagement activities** | **Who involved** | **Type of community engagement in the research process** | **Outcome** |
| --- | --- | --- | --- | --- | --- |
| Agot et al. 2019  Kenya and South Africa | - Engaging study participants in interpreting results: lessons from the study that assess the preferences for attributes of tablets, vaginal rings, and injectable products for dual prevention of HIV and pregnancy (TRIO Study) | - Former participants of the TRIO study attended five study dissemination sessions and five one-on-one sharing sessions six months after exiting the study | - 40 % of former TRIO study participants | - Interpreting study findings   ***A4, A5/B2*** | - The dissemination sessions provided an important forum for study participants to interrogate and explain the results to minimize possible misinterpretation. - This exercise helped give context to the results ensured correct lessons were derived from those results and increased credibility of the findings reported by the investigators. |
| Angwenyi et al. 2014  Kenya | - Community engagement for a pediatric randomized controlled malaria vaccine trial | - Consultation and sensitization with district stakeholders (Month 1) - Community entry and sensitization of stakeholders in the three sites (Months 2 to 6) - Identification and recruitment (Months 8 to 27) - Follow-up of research participants (Starting from month 8) - Continuous feedback to and from community (Starting from month 8) - Feedback of results, for example, preliminary study results disseminated (Starting from month 8) | - District Medical Officer of Health and District Health Management Team at Kilifi District Hospital. - All health facility in-charges working in Kilifi District - Dispensary health committees, dispensary staff and community health workers (CHWs) - Local administration: District officers, local (assistant) chiefs, and village elders - Others: primary school head teachers, religious leaders, a local District Stakeholders Forum - CHWs and fieldworkers - Fieldworkers and other key gatekeepers - Involves all of the above | - Community entry/   Sensitization   - Recruiting study participants - Interpreting study findings - Giving/ attending presentations at meetings and conferences - Implementing the intervention     A2, A3, A4, A5/B2 | - Cleared pre-existing concerns and misconceptions; increase visibility, awareness and trust in the trial staff - Increase enrollment and retention rate |
| Bandewar, Kimani and Lavery 2010  Kenya | - The community engagement practices of the Majengo Observational Cohort Study (MOCS) that examined sexually transmitted infections, in particular HIV/AIDS, in a cohort of sex workers | 3 distinct phases of community engagement in the MOCS:   1. Reaching out: mobilization, dialogue, and education using experienced persons from the community 2. Foundations of trust through relationships of care; and 3. Leveraging existing social capital to form a cohort community. | - Community health expert - Research staff and Majengo Clinic (MC) staff, and research cohorts - both the MC team and the cohort members | - Community entry/   sensitization   - Recruiting study participants   ***A2, A3/B1*** | - The findings demonstrate the importance of community participation in research and the accompanying gains in personal security and solidarity that have kept the women in the cohort, some for 20 years or more. |
| Beard et al. 2020  South Africa | - Challenges of developing a district child welfare plan in South Africa: lessons from a community-engaged HIV/AIDS research project | - Field office opened and community members hired as research assistants and office administrators (startup) - Principals and teachers from local schools assist with the identification of orphans and children at risk of being orphaned and their caretakers are invited to participate in the study (startup) - Establish a community advisory committee (startup) - Engage the community in an ongoing discussion about the study purpose and procedures (promote project visibility and local ownership; input elicited on survey instruments, and data collection) - Conference advisory council initiated and three rounds of data collection findings presented for different stakeholders including the study participants - Communicate preliminary research findings from three rounds of data collection to the general community - Produce and distribute advocacy communications to facilitate behavior change at the district level | - District Departments of Education (DoE), Health (DoH), and Social Development (DoSD) NGOs; CBOs; local businesses, other private sector stakeholders | - Community entry/Sensitization - Recruiting study participants - Designing research tool/format - Interpreting study findings - Giving/ attending presentations at meetings and conferences   ***A2, A3, A4, A5/B3*** | - The project was transparent about study goals, communicated frequently with community collaborators to understand local concerns about the research, and engaged in ongoing review and modification of engagement strategies including data collection tool revisions. - By the end of study period, collaboration and coordination among local social service organizations had increased substantially. |
| Broder et al. 2020  Africa Vs Americas/Switzerland | - Standardized metrics or strategies for community engagement and recruitment in HIV prevention trials i.e., HIV Vaccine Trials Network (HVTN) and the HIV Prevention Trials Network (HPTN) across different regions | - The Networks support partnerships between communities and researchers throughout the entire research process and engage with communities at the research sites where studies are conducted as well as with other key internal and external stakeholders through regular tele/video conferences, protocol team meetings, site assessment visits, study-specific consultations, and training, and workshops. - Community engagement activities were done on three levels:  1. **Protocol-specific level**: community working groups provide guidance to the study protocol teams; provide input into protocol development; adapt sample consent forms for local use and develop other study-related materials; increase the capacity of members through participation in protocol-specific training and regional workshops; inform strategies for recruitment and retention; and assist in monitoring any emerging issues in the community 2. **Site-specific level**: community advisory boards (CABs) serve as the voice for the community and study participants in a particular locale. CABs bring specific, unique expertise to the research process by participating in defining the scientific agenda and informing researchers of local issues or concerns that can affect the conduct and successful implementation in that locale. 3. **External stakeholder level**: Community representatives contributed to the development of local recruitment plans and identify the recruitment strategies | - Community working groups composed of one community educator and one community advisory board representative from each participating site - CABs - Site community engagement staff - External stakeholders include community advocates and civil society groups such as pastors, traditional healers, tribal chiefs, Ministry of Health representatives, community-based organizations, and others | - Concept development (defining the problem) - Developing sampling procedures - Designing interviews and/or survey questions or any research tool or formats - Recruiting study participants - study implementation, and - results dissemination   ***A1, A2, A3, A4, A5/B4*** | - Through consultation with community representatives from each clinical research site recruitment strategy descriptors developed and effectiveness of recruitment strategies varies by region - Globally, referrals were the most efficient and effective recruitment strategy. Print materials were also valuable globally. - In Africa, in-person outreach was quite effective while Internet strategies had better success in the Americas/Switzerland than in sub-Saharan African countries |
| Corneli et al. 2007  Malawi | - Involving communities in the design of clinical trial protocols on the safety and efficacy of antiretroviral and nutrition interventions to reduce postnatal transmission of HIV | - Community members were involved in formative research to inform study protocol so that to modify the protocol in a way that helps to achieve cultural acceptability while maintaining study objectives including the informed consent process | - HIV-positive mothers, mothers of undisclosed HIV status, pregnant women, grandmothers of grandchildren, fathers who have infants less than 1-year-old, Health providers, traditional birth attendants, and community leaders | - Protocol design   ***A1/B2*** | - Incorporating formative research into protocol helps to modify the protocol and ensures participant understanding of the research, safeguards participants and increases feasibility and acceptance of the clinical research in the community |
| Davies et al. 2012  Uganda | - Design user-friendly adverse event reporting forms to capture information on events associated with artemisinin combination therapies (ACTs) for the treatment of malaria using a participatory approach | - A participatory method was used to develop a new reporting form that engages the target audience (community members) in the problem-defining and solving process. - The project had three phases namely:  1. Review of existing forms- To Understand how current pharmacovigilance reporting forms were being used in Uganda. The forms were reviewed in two focus group discussions with community medicine distributors (CMDs) and then health workers, in which participants were encouraged to discuss their experiences with anti-malarial treatment and reporting of AEs with existing forms and procedures. 2. Form development- Draft form was produced for passive reporting. Two participatory workshops held, one with CMDs and one with health workers. A local artist drafted and refined sketches at the request of participants. 3. Pretesting, which involved one observation phase with CMDs and a formal testing phase with graduate fieldwork recruits and CMDs. | - Community medicine distributors, health workers, malaria surveillance project field workers - The field team consists of two local artists who draft and refine the sketches at the request of participants in an understandable way by low-literacy | - Problem-defining and solving process   ***A1-A5/B4*** | - Developed novel adverse event reporting forms that can be used by non-clinicians to capture pharmacovigilance data for anti-malarial drugs. |
| Denison et al. 2017  Sub-Saharan Africa | - Youth engagement in developing an implementation science research agenda on adolescent HIV testing and care linkages in sub-Saharan Africa | - Youth living with HIV (YLHIV) participated in a two-day implementation science research agenda setting on a project called Supporting Operational AIDS Research (Project SOAR). The young people shared their views and experiences at the meeting and voted on the priority research questions across all working group topics. | - Youth living with HIV from SSA, funders, researchers, programmers | - Research agenda setting   ***A1/B2*** | - Youth participation influenced working group discussions and the development of the implementation science agenda.      - The meeting participants developed the research agenda to guide future implementation science research to improve HIV outcomes among adolescents in sub- Saharan Africa. |
| Diallo et al. 2005  Mali | - Community permission to conduct medical research (malaria vaccine study) | - The research team identified individuals who could be considered legitimate representative of the community and involved them in different meetings. Modification of the protocols and consent form was done based on the feedback given from the community during the meetings | - Community leaders (health authorities, neighborhood chiefs, school authorities, religious leaders, chief district administrator and mayor) - Traditional health practitioners | - Community entry/permission - Research implementation   ***A1, A2, A3/B2*** | - The process (6 steps) of obtaining community permission both initiated and facilitated the process and disclosure for individual informed consent. |
| Dierickx et al. 2018  Gambia | - Community sensitization and decision- making for malaria transmission dynamics study and prinogam malaria trial | - The fieldworkers and nurses were at the interface between the study team and community members, both during the community sensitization and individual informed consent process - Community members attended the community sensitization meetings - The information given during the community sensitization meetings was consistent with the information written down in the consent form and information sheet | - The field team (field workers and nurses) - Key community members (representatives) | - Community sensitization     ***A3/B1*** | - Although not substituting individual consent, community sensitization meetings represent a first step in getting access to the communities and improve the community engagement and informed consent process |
| Doshi et al. 2017  Kenya | - Contextualizing the willingness of men who have sex with men (MSM) to participate in HIV vaccine efficacy trials | - Community researchers who were respected leaders in their communities were selected from their respective MSM communities. These were experienced in sexual health research and programming and played a central role in the design of data collection tools, collection of data by conducting qualitative interviews and data analysis. | - Community | - Design of data collection tools - Data collection - Implementation - Data analysis   ***A2, A3, A4/B3*** | - Willingness to participate in HIV vaccine efficacy trials was highly motivated by various forms of altruism. Specific researcher responsibilities centered on safe-guarding the rights and well-being of participants was found to govern willingness to participate in future preventive HIV vaccine. |
| Faye and Lugand, 2021  Democratic Republic of the Congo, Nigeria and Mozambique | - Participatory research for the development of information, education and communication tools to promote intermittent preventive treatment of malaria in pregnancy (IPTp) | - The researchers interacted directly with community participants on the field, by listening to them, and by collecting their opinions on how tools could be improved | - Graphic designer - Pregnant mothers - Community relays - Health workers | - Implementation intervention   ***A3/B2*** | - Discussion with community participants helped to understand their opinions, update the communication materials by anticipating the user needs - IEC tools were developed in a way that are user friendly and adapted to social contexts using an iterative and collaborative process |
| Folayan et al. 2019  West Africa | - Priorities for community engagement for research conducted during infectious disease outbreaks (Ebola) | - Trained expert laypersons who were members of ethics review committees represent the interests of the community participated in the consensus reaching process in the four phased consultative meetings using Delphi method. - Expert laypersons participated in two rounds of preliminary face-to-face discussions with research team; review of summary documents; and participated in the development of consensus document based on their feedback till final revision that focused on the considerations of community engagement in research design and implementation during infectious disease outbreaks | - bioethicists, social scientists, researchers, policy makers and laypersons who work with ethics committees - guidelines for [stakeholder engagement in] biomedical HIV prevention trials | - Priority setting/agenda setting   ***A1/B2*** | - Perspectives of expert laypersons who were members of the ethics review committee were considered in the Good Participatory Practice for Trials of Emerging (and Re- emerging) Pathogens (GPP- EP) guideline for stakeholder engagement in biomedical prevention trials. - Community engagement is an ethical imperative for clinical trials conducted during infectious disease epidemic outbreaks like the recent Ebola epidemic. Clinical trial research in such a context helps to facilitate a collaborative process that both enhances the conduct of the clinical trial, and leaves the community better off. |
| Freudenthal et al. 2006  Tanzania | - Participatory action research in primary schools in order to create enabling environments for the schoolchildren and other community members to adapt practices relevant for reducing the transmission of schistosomiasis | - Researchers from Sustainable prevention of endemic schistosomiasis (SPES) project established good link with community members and approached the school director and village leaders to ensure broad participation of the community members. Their engagement of the community is described below:  1. First community leaders organized meetings with schools and community 2. A variety of school activities organized in part to mobilize different stakeholders to reflect and participate in actions (screening and treatment of schoolchildren for schistosomiasis and intestinal helminthes; development of slogans for prevention; write school essays; prepare video recorded drama, songs and dances; and household sanitation survey) i.e., pupils were involved in the household survey as researchers and change agents 3. Feedback meetings to reflect on results in schools and larger community 4. Community members decide to create safe swimming places 5. Reflection of the household sanitation survey as part of the school activity 6. Teachers develop curriculum for schistosomiasis education in primary schools | - The SPES research team (comprising of medical health professionals and social scientists from Tanzania, and two European countries) - The Tanzanian research team (one medical doctor, several community health nurses and laboratory technicians) - Representatives from Referral hospital and schools, village leaders, and community members | - Community sensitization - Defining/identifying the problem - Designing interviews and/or survey questions or any research tool or formats - Recruiting study participants - Collecting data - Analyzing collected data - Interpreting study findings - Implementing the intervention   ***A1, A2,A3, A4, A5/B5*** | - Many methods can be effective in school-based programs, but they must involve reflection and feedback loops in order for effective learning to take place. The breakthrough in this project came when the teachers shifted their perspective from “helping the researchers with their project” to seeing themselves as active participants and stakeholders in the prevention of schistosomiasis. - They have now witnessed that schistosomiasis is highly endemic in the area and that they as teachers are responsible for doing something about it in the schools |
| Hartley et al. 2021  Mali | - Technology ‘co-development’ (between researchers, stakeholders and local communities) by those developing gene drive mosquitos for malaria eradication. | - The malaria consortium involves collaborative partners in Africa, Europe, and North America and was organized into science, regulatory affairs, project management, communications and stakeholder engagement. The Malian engagement team was responsible for working with stakeholders at the local, national and regional level and undertakes community engagement around its locality and shares short videos and materials about its activities | - Researchers - Stakeholders - Local communities | - Community sensitization   ***A3/B1*** | - For Malians, co-development reflected Mali’s broader socio-political context and a desire for African scientific independence and leadership. It was mobilized to secure community and stakeholder support for gene drive mosquito field trials, through outreach, building local scientific capacity and developing those institutions (e.g. regulatory) necessary for field trials to go ahead. |
| Hullur et al. 2016  South Africa | - Community–based participatory research (CBPR) methods to identify community perspectives on HIV, violence and health surveillance | - The Heath and Socio–Demographic Surveillance Site (HDSS) established the Learning, Information, dissemination and Networking with Communities (LINC) group to enable community participation in research and governance. LINC enhances research quality through the feedback of research results to community stakeholders. - The research adopted a community–based participatory research (CBPR) approach and communities participated in identifying and defining health problems only. - LINC staff then approached different stakes to convene discussion groups that broadly represented the community - Then selected community members participated in group discussions and share their collective perspectives on the relationships between medical problems and their social and health systems determinants | - Women of reproductive age (WRA), family members, traditional healers, religious leaders, community health volunteers, health workers, village officials, and community leaders in villages | - Identifying and defining health problems   ***A1/B2*** | - The discussions on HIV/AIDS revealed serious problems with respectful care, confidentiality and patient dignity - The surveillance system was also discussed and the community views on the surveillance was largely positive reflecting established public engagement and suggested some modifications to ensure that surveillance is respectful of loss, grieving and mourning, and for confidentiality and sensitivity when discussing deaths of relatives at the individual level |
| Kamanda et al. 2013  Kenya | - Adaptation of community-based participatory research (CBPR) approaches to conduct orphaned and separated children’s assessments related to their health and wellbeing (OSCAR), HIV/AIDS related research | - The study has engaged community members both for the community and research team in four phases:  1. Phase One: During problem identification, planning, feasibility and evaluation  - Key community members were involved in identifying the problem and identified key questions and priorities; give feedback to the protocol; participate in the community meetings arranged by chiefs and village elders regarding the feasibility of the project and contextualizing to local procedures and cultural context; - CAB reviewed *mabaraza* feedback and approved study procedures and data collection instruments  1. Phase Two: Sampling and recruitment  - Community health workers (CHWs) integrated into the research team - Assistant chiefs and village elders identify households caring for orphaned and separated children - Community meetings with orphaned caregivers  1. Phase Three: Retention, validation and follow-up  - Monthly meetings - Annual mabaraza - Quarterly CAB meetings - Ongoing community feedback and input to the project  1. Phase Four: Analysis, interpretation and dissemination  - CAB quarterly meetings with the research team to review preliminary findings, discuss interpretation within the local context and determine appropriate dissemination strategies - The project directly disseminates information semi-annually to the Children’s Services Forum and attends their monthly meetings - Disseminate information directly to the community through the CHW’s to gain in- valuable feedback and ensure the on-going cultural and community relevance - Presentations of the data in lay format through mabaraza | - County administration, Chief and village elders, traditional community (mabaraza) - CAB (Village Elders, Assistant Chiefs, Chiefs, opinion leaders, representatives of Charitable Children’s institutions, District children’s officer, and representatives of the orphaned children in the County, other relevant stakeholders and the project co-investigators.) - CHWs | - Community entry - Problem identification - Developing sampling procedures - Recruiting study participants - Retention, validation and follow-up - Analyzing data/review preliminary findings - Interpreting results - Dissemination   ***A1, A2, A3, A4, A5/B4*** | - By collaborating with the community and stakeholders from research question development, through to implementation and dissemination of results, epidemiological and public health research projects in sub-Saharan Africa have the potential to increase the validity and generalizability of the research. - The on-going contribution of the community in the research process has been vital to participant retention and data validation while ensuring cultural and community relevance and equity in the research agenda. |
| Mabunda et al. 2016  South Africa | - A Community-Based Participatory Research (CBPR) approach to needs assessment for adapting TB directly observed treatment (DOT) intervention program | - Professional nurses, DOT supporters, community members and patients were invited to participate in a meeting workshop and focus groups in the study - The planning team comprised of TB coordinators (provincial TB managers, district TB coordinators) involved in recruitment of participants and conducting focus groups. - Participants identified the barriers and facilitators to health-care seeking and adherence to treatment, and the strategies and messages that inform the design of an adapted intervention program | - TB coordinators, professional nurses and DOT supporters - Patients and community members | - Recruiting study participants - Collecting data - Implementing intervention   ***A2, A3/B3*** | - The adoption of participatory approach involving various stakeholders to develop an intervention to meet the community needs |
| Marsh et al. 2011  Kenya | - The role of community in international collaborative biomedical research (community perceptions in genomic epidemiological study and malaria vaccine trial) | - Community engagement was supported by a centralized group of full-time community facilitators and draws on action research principles of continuous evaluation and adaptation - The community engagement activities are summarized below:  1. Public engagements through schools, public meetings and events in the community, regular interactions with opinion leaders who are in continuous contact with the wider community were done to strengthen the general awareness and understanding of biomedical research concepts and activities 2. Public meetings and participatory workshops that support the interactivity between researchers and community to promote visibility, accountability, reliability and perceived fairness were conducted to build appropriate level of trust 3. Meetings with community representatives (community members and opinion leaders) conducted to support consultation/ deliberative discussions and to understand how general or specific research project activities may interfere with freedom of choice 4. Small scale meetings with invited groups (including field workers and driver) were done to build trust on specific studies 5. Meetings/discussions with ‘typical’ representatives and opinion leaders conducted to understand how research activities may generate ‘hidden’ costs or benefits; and to ensure validity of science | - Opinion leaders, community leaders, government health staff, project staff, field workers, drivers, community members | - Community entry/sensitization   ***A3/B2*** | - Taking individual informed consent seriously involves understanding and addressing the influence of communities in which individuals’ lives are embedded. - Individual participation can generate risks and benefits for communities as part of the wider implications of research |
| Meiring et al. 2019  Malawi | - Community engagement before and during of Typhoid Conjugate Vaccine Trial | The community engagement activities are summarized below:   - A community engagement taskforce designed and implemented engagement activities for trail initiation - Community advisory groups participated in community representative meetings and consulted on engagement plan and study activity - Study protocols shared with ministry of health and education and study information was disseminated to each school via presentations, discussion groups, flyers, posters and letters home - Community leaders meeting held to explain about the study - Individual school-based meetings held to present a study overview and seek participation to establish vaccine clinics on schools’ property - Community health committee meetings held focused on their assistance in disseminating study information and relaying questions and feedback from the community to the study team - Mobile Van with audio recording informing people and inviting them to vaccine clinic. A local musician created a jingle containing study messages and invited guardians to bring children for vaccination. This jingle played via a mobile van with large speakers on the roof that drove through the communities. | - Community engagement taskforce (consisted of education officers, school health and nutrition officers, EPI officers, health promotion officers, the chief nursing officer, DHO administration and MLM staff) - Community advisors - MoH/MoE - Community leaders - primary education advisors, teachers, parent-teacher association, school management committee members, and women’s groups - Health surveillance assistants (HSAs) and village health committees - Local musician | - Community entry/sensitization   ***A3/B2*** | - Community engagement was a benefit to the trial and encouraged community recruitment in a number of ways  1. Providing feedback that helped design engagement activities and consent procedures 2. Create awareness and increase recruitment 3. Create opportunity to provide information and answer questions 4. Promote policy interest and future rollout |
| Morin et al. 2008  Zimbabwe | - Evolution of community advisory boards (CABs) and community partnerships at international research sites conducting HIV prevention trials | - The relationship between research and community has been greatly facilitated through the work of the Community Liaison Department, where staff oversees the interface of research with the CAB and the community at large. The Department ensures that appropriate constituencies are represented in the CAB, educates CAB members about CAB functions, delivers protocol related trainings, and fosters active CAB participation in the research. - CAB representation is broad based and their accountability is to the community - The responsibilities of CAB have evolved beyond the intermediary role between research and community - CAB helped identify and implement methods to locate lost participants and achieved full retention rate | - CABs | - Community entry/sensitization   ***A3/B1*** | - The presence of research and the establishment of community partnerships via the CABs were a factor in increasing HIV knowledge, awareness, and testing - CABs expanded their original function and became advocates for broader community interests beyond HIV prevention |
| Mtove et al. 2018    Sub-Saharan Africa (Benin, Kenya, Malawi, Tanzania, and Uganda) | - Multiple-level stakeholder engagement in malaria clinical trials (multicenter clinical trial evaluating intermittent preventive treatment of malaria in pregnancy, IPTp) | Level of engagement was at different levels with multiple level stakeholders including the local community   - At International level multiple stakeholders were engaged and had an influence in study design, research ethics and policy development - At national and district level different stakeholders such as the government departments, ministries of health, and ethics committee were engaged and has an influence in regulatory requirements, standards of care and research ethics - At local community level different stakeholders such as the community, family and study participants were participated. Community engagement measures undertaken by investigators included local meetings with community leaders to explain the research aims and answer questions and concerns voiced by the community (consent, perception, values and customs). The investigators also engaged with family members of prospective trial participants in order to be sensitive to local practices and beliefs. | - International stakeholders - National stakeholders (including the health ministers and regional/local community health workers) - Community (village leaders, elders, and religious leaders), Family, and Study participants | - Community entry/ sensitization - Study design   ***A2, A3/B2*** | - Engagement with key stakeholders at international and national levels enabled the Sponsoring Entities to address challenges by aligning the study design with the requirements of health and regulatory agencies and to understand and address healthcare infrastructure needs prior to trial initiation. - Local stakeholder engagement, including community members, study participants, and family enabled the investigators to address challenges by ensuring that study design and conduct were adapted to local considerations and ensuring accurate information about the study aims was shared with the public. |
| Nakalega et al. 2021  Uganda | - Ethical considerations for involving adolescents in biomedical HIV prevention research | - CAB recruit adolescent girls and young women (AGYW) - AGYW attended a stakeholder consultative meeting aimed to create awareness about the study, gain community insights about the study, and learn about community perspectives of engaging adolescents younger than 18 years (the legal age of consent) in sexual reproductive health related research | - CAB - AGYW, adult participants including local council leaders, youth organization heads, community youth leaders, medical professionals, women representatives | - Community entry/sensitization   ***A2, A3/B2*** | - Stakeholder consultative meetings held prior to trial implementation improved study conduct in multiple ways. - Transparent and mutually respectful stakeholder engagement with diverse community representation was critical to gaining a richer understanding of community perspectives and concerns about female adolescent participation in HIV research. |
| Nakibinge et al. 2009  Uganda | - How a research project on HIV epidemiology in rural Uganda has engaged the community over the past two decades | - A community project advisory board initially played a key role as community liaison - Community consultation and feedback was subsequently assured through the local counsel (LC) system of civic administration thereby facilitating sustainability and community acceptance - The project engaged not only the formal LC community leaders but also informal leaders (individuals with considerable influence on community opinion by virtue of their status or reputation). - An influential informal leader was recruited as the first community liaison officer, and other informal leaders have been recruited as advisers and guides during annual survey rounds | - CAB - Local counsel (LC) - Community leaders - Community representatives | - Community entry/ sensitization   ***A3/B2*** | - There was a feedback system for identifying problems in the community, generating solutions for corrective action and planning (in consultation between the project and community representatives) and reporting back on implementation of these solutions - Dialogue with informal leaders established a good relationship built on trust, transparency, integrity and commitment. An |
| Nyika et al. 2010  Burkina Faso, Mali and Tanzania | - Engaging diverse communities in malaria vaccine trails | - The general steps followed in engaging communities at various sites were as follow:  1. Ethical and regulatory approval of the intended clinical trial in each country 2. Administrative approval from local government structures such as local district health offices 3. Permission to enter the community: (community leaders approached to initiate engagement with the community) 4. District community engagement facilitated by the community leaders 5. Meeting with ordinary members of community including heads of families which served to inform, sensitize and invite potential participants 6. Recruitment of individual participants | - National and district health offices - Community leaders - Community representatives - Participants | - Community entry/   Sensitization   - Participant recruitment   ***A2, A3/B2*** | - Community engagement enables two-way sharing of accurate information and ideas between researchers and researched communities, which helps to create an environment conducive to smooth research activities with enhanced sense of research ownership by the communities |
| Olaseha and Sridhar 2005  Nigeria | - Participatory action research: community diagnosis and intervention in controlling urinary schistosomiasis | - An intervention planning network was created for designing and implementing effective interventions - The Department of Health Promotion and Education of the University of Ibadan with the students and specialist staff took the leadership role while other agencies involved carried out supportive roles. - The parents and teachers of the pupils affected were contacted and adequately informed at their various PTA meetings. - A group of students (Advanced Diploma in Health Education) and staff of the Department of Health Promotion and Education were held to sensitize the communities and teachers about the nature and the challenges of the problems. - A Ward Health Council (WHC) was formed to represent communities and work and collaborate with the created network (team) - The involved mass media worked relentlessly to sensitize residents of Ibadan metropolis about the outbreak of schistosomiasis in the study communities as part of the Information Education Communication (IEC) strategy. - The community members in Ibadan felt that Schistosomiasis is their major health problem and listed it in their priority list and they systematically pursued the relevant authorities in controlling the disease. | - Oyo State Ministry of Health, Oyo State Water Corporation, Ibadan North Local Government Area, the Oyo State Family Support Program, Broadcasting Corporation of Oyo State, Nigerian Television Authority, Radio Nigeria Ibadan, the Directorate of Food, Roads and Rural Infrastructure (DFRRI), the Health Committee of the study community, Parents Teachers Association (PTA) of various schools, and office of the Governor of Oyo State | - Problem identification and agenda setting - Designing an intervention plan - Community entry/Sensitization - Implementing the intervention     ***A1, A2, A3/B3*** | - The communities at the end enjoyed improved water supply and reduced their exposure from the stream, which was the source of infection. All actors benefitted from the learning experiences and the skills, which the participatory action process offered. |
| Pare Toe et al., 2021  Burkina Faso | - Engagement activities relevant to field trials on non-gene drive genetically modified - mosquitoes as well as an assessment framework (Malaria) | - Stakeholder identification and prioritization - Demonstrated an extensive mapping and understanding of stakeholders at all the levels. - At the village level, the understanding was gained from ethnographic studies that highlighted “gate-keepers” in the community, official and non-official power structures, dominant and minority social groups and also the quality of relationship between village leaders and administrative authorities - The engagement audit checklist that revolved around the following themes: Identification and analysis of stakeholders, Information, Consultation, Negotiation and partnership, Complaint management, stakeholder involvement in project monitoring, Feedback to stakeholders, and Management functions. | - leaders, women, men, young people, administrative authorities and public servants and minority ethnic groups | - Community entry/Sensitization   ***A3/B2*** | - The stakeholder engagement process, including the assessments, was crucial to building trust and empowerment with directly affected communities and other key stakeholders. - These learning will be critical for the project’s next steps of engagement with communities, stakeholders and the broader public. They demonstrate the importance of responsibility and accountability mechanisms that can provide public confidence in how the project has been engaging communities and stakeholders with the appropriate respect for their autonomy and deliberation process. |
| Reddy et al. 2010  South Africa | - Functions and operations of Community Advisory Boards (CABs) in HIV/AIDS vaccine trials. | - Recruited youth by consulting staff of institutions working with youth (government offices, churches, non-governmental organizations, HIV comprehensive care centers, compassion homes that care for disadvantaged children and schools). - As a part of a larger research project on the effects of HIV testing and disclosure in the context of a research study on adolescent behavior and psychosocial wellbeing; to solicit community feedback and buy-in and to inform protocols for the parent study an Adult Community Advisory Board (CAB) and Youth Advisory Board (YAB) were created to examine stakeholder perceptions about recruitment, informed consent, HIV testing and disclosure of results, and compensation/inducements for HIV-related studies involving adolescents. | - Health and other professionals engaged with adolescents, caregivers/parents, and adolescents | - Community sensitization - Research design - Recruiting study participants   ***A2, A3/B2*** | - CABs are seen primarily to serve and be accountable to the community on the one hand or to the trial site and the researchers on the other. - Generally, this paper came with four themes named 1) Purpose (protect community interests and advance research goals) 2) membership and representation 3) power and authority, and 4) source of support and independence |
| Rennie et al. 2017  Kenya | - Significance of benefit perceptions for the ethics of HIV research involving adolescents | - Recruited participants for both the Community Advisory Board (CAB) and Youth Advisory Board (YAB) FGDs by consulting staff of institutions working with youth | - Staff of institutions working with youth | - Recruiting study participants   ***A2/B2*** | - Study participants regarded participation in research as largely beneficial for individual study participants and their communities. - Only a few participants suggested that research participation is not straightforwardly beneficial. |
| Shahmanesh et al. 2021  South Africa | - Community Based Participatory research to iteratively co-create and contextually adapt a biosocial peer-led intervention to support HIV prevention | - Community Leaders selected peer-navigators from their own community - Peer navigators underwent 20 weeks of training - The peer navigators discuss the vignettes and co-create the Thetha Nami (`talk to me’)- an intervention which included peer-led health promotion to improve self-efficacy and demand for HIV prevention, referrals to social and educational resources, and accessible youth-friendly clinical services to improve uptake of HIV prevention - They refined Thetha Nami intervention to add three components: structured assessment tool to tailor health promotion and referrals, safe spaces and community advocacy to create an enabling environment, and peer-mentorship and navigation of resources to improve retention in HIV prevention. | - Community Leaders, Selected community member as peer-navigator | - Community entry/   Sensitization   - Recruiting study participants - Interpreting study findings - Giving/ attending presentations - Implementing the intervention   ***A2, A3, A4, A5 /B4*** | - Peer-navigators critically engaged with vignettes, brainstormed solutions and mapped the components to their own intervention implementation area - The intervention components were plotted to a Theory of Change which the peer-navigators refined. - The peer-navigators approached 6871 young people, of whom 6141 (89%) accepted health promotion and 438 were linked to care. |
| Silumbwe, Halwindi and Zulu 2019  Zambia | - Community engagement strategy’s role in shaping participation in mass drug administration programs for lymphatic filariasis (LF) | - The selection of In-depth Interview participants was guided by the program implementers and community members | - Community members, traditional leaders, and program managers | - Recruiting study participants   ***A2/B2*** | - Facilitating participation in Mass Drug Administration for LF requires designing and implementing effective community engagement strategies that take into account local context. |
| Simwinga et al. 2016  Zambia and South Africa | - Community Engagement for HIV Combination Prevention strategy: Lessons Learnt from Community-Randomized Study | - In Zambia, a national consultative meeting was held as a starting point for subsequent meetings. - In South Africa, potential study communities were identified in dialogue - Prior to the study, ‘Broad Brush Surveys’ (BBS) were carried with the aim of understanding the communities. The findings, combined with iterative discussions between the social science, intervention and CE teams, informed the development of strategies for community representation. - The suggestions provided by the CAB were recognized and used for protocol and tool revision as well as intervention message development | - Community and government representatives, former community Advisory board (CAB) members - Provincial and local Department of Health representatives and prominent advocacy groups such as the Treatment Action Campaign | - Community entry/sensitization. - Designing research tool/format - Implementing the Intervention   ***A2, A3/B2*** | - BBS findings provided information on alternative (e.g. herbal or traditional) treatment options for HIV and other ailments and, based on these findings, it was decided to include traditional healers on study CABs. - Community consultations and BBS activities formed the basis not only for understanding the community, but also for collecting vital information to inform development of engagement tools. |
| Tarr-Attia et al. 2018    Liberia | - Community informed research on malaria in pregnancy | - Offered training in basics of medical research ethics to a group of traditional community representatives. All trainees were invited to constitute a Community Advisory Board (CAB). - Prior to implementing the study, advice from the CAB was sought with regards to the appropriateness of the informed consent and data collection procedures. | - Pregnant women, traditional community representatives, and hospital staffs | - Community entry/sensitization   ***A2, A3/B2*** | - In spite of generalized distrust towards the health establishment, pregnant women might be interested in participating in malaria research, provided this is perceived as useful and that it is planned, in collaboration with the communities, to address their most immediate prevention and care needs. |
| Vreeman et al. 2012  Kenya | - Understanding the community perspective using traditional community assemblies on the participation of vulnerable children in biomedical research (HIV related) | - The researcher used the existing traditional community assemblies called mabaraza (it is used for both sharing information and gathering community opinions on issues). - The mabaraza were officially organized by a Chief or Assistant Chief, who invited village elders and asked them to bring at least one caregiver of an orphaned or separated child, in addition to inviting their village community | - The community group engaged include: - Chief, Assistant chief, village elders, caregivers and other community members. | - Community entry/sensitization, Consent process   ***A2, A3/B2*** | - Positive attitudes towards involving vulnerable children in research, largely because they assumed children would directly benefit |
| Yotebieng et al. 2019  Sub-Saharan Africa | - Developing consensus around research priorities for the Treat All policy (the treatment of all people with HIV, irrespective of disease stage or CD4 cell count) implementation in SSA | - A set of research priorities was collectively formulated and refined by a technical working group and shared among stakeholders for review, deliberation, and prioritization. A round of Delphi techniques applied | - researchers, - Implementation experts, policy/decision-makers, and HIV community representatives | - Research Agenda setting     ***A1/B3*** | - The process resulted in a list of nine research priorities for generating evidence to guide Treat All Policies, implementation strategies, and monitoring efforts. - The resulting research priorities highlight important evidence gaps that are relevant for ministries of health, funders, normative bodies, and research networks. |

**NB:**

**A: Categories of community engagement in the research stages/processes (A1-A5)**

- **A1=Conceptualization:** community engagement in defining or identifying the problem, prioritizing questions, setting objectives and background of the research
- **A2=Research Design**s: community engagement in choosing research methods, sampling, designing interviews and/or survey questions or any research tool or formats and recruiting study participants
- **A3=Research Implementation:** community engagement during community entry or study initiation and sensitization, collecting data, supervision, implementing interventions, monitoring, quality control etc.
- **A4-=Data analysis and interpretations:** community engagement in analyzing data, interpreting findings, writing reports
- **A5=Disseminations and translations:** community engagement in presentations, workshops, etc.

**B: Level of engagement of the community in the research process (B1-B5)**

- **B1=Inform**: Researchers provide communities with information so that the community is kept informed (one way)
- **B2=Consult**: Researchers obtain specific types of input or feedback from the community so that the community members are kept informed, listened to and given feedback on how their input influenced the study process
- **B3=Involve**: community members to work directly with researchers in conducting the study so that the community members ensure that their views are understood and considered
- **B4=Collaborate:** Partnering community members with researchers throughout the process of conducting the research, community members are looked for advice which will be incorporated in the final decision
- **B5=Empower**: giving over control or delegating of conducting some or all part of the study process, community members have the power to give final decisions
