# Supplementary material for: Community engagement in research addressing infectious diseases of poverty in sub-Saharan Africa: A qualitative systematic review
Source: PLOS Glob Public Health. 2024 Jul 15;4(7):e0003167. doi: 10.1371/journal.pgph.0003167 (PMC11249264; doi:10.1371/journal.pgph.0003167)
Supplement: S4 Table — (DOCX) [file pgph.0003167.s007.docx]

**S4 Table**: Summary of findings related to challenges and their mitigation strategies on community engagement in research

| **S.No** | **Study reference** | **Barriers/challenges/concerns** | **Mitigation measures undertaken** |
| --- | --- | --- | --- |
| 1 | Angwenyi et al. 2014 | - Some community leaders attempting to exert pressure on people to enroll; local wording in information sheets and consent forms feeding into serious anxieties about the trial; and concerns about reduced CE over time | - Negative effects of these challenges were mitigated through changes to ongoing CE activities, and final information sharing and consent being conducted individually by trained clinical staff. |
| 2 | Beard et al. 2020 | - The project was less successful in maximizing opportunities for stewardship and shared control by the community - Communicating study results in a timely and audience-appropriate manner was another challenge - Tensions were posed by the university-community partnership – two universities were equal intellectual partners in the study design; however, the community was not a third, equal partner in determining study questions or methods | - Early initiation of engagement activities; careful characterization of the community and it's changing needs; establishment of trust with stakeholders; and development of community assets - The challenge of timely dissemination of results was solved by one of the universities by doing secondary data analysis and publishing data through reports and white papers. |
| 3 | Broder et al. 2020 | - There were challenges related to the representativeness of community members for the broader community, and the impartiality of advisory community structures funded by clinical research sites | - Consultations with communities helped to conduct recruitment that was sensitive to avoid stigmatizing populations that were deemed at increased risk for the acquisition of HIV. |
| 4 | Dierickx et al. 2018 | - In practice, everyone may not be present in community meetings | - Key figures within the compound are more likely to be present and explain the study to compound members, acquaintances, and friends afterward |
| 5 | Doshi et al. 2017 | - In addition to individual and socio-cultural motivators and barriers, both perceived benefits of a future HIV vaccine and experience with/ knowledge of medical research were found to influence their willingness to participate | - Strategies towards community engagement and recruitment of trial volunteers should be informed by analysis and understanding of all three influences (i.e., individual/socio-cultural motivators/barriers, future vaccine acceptability, and experience with/knowledge of medical research) individually and in relation to one another. |
| 6 | Kamanda et al. 2013 | - Eliciting community feedback through multiple sources was a challenge | - In order to address the challenge of responding to the needs and requests of the community, the project has taken time to listen to the communities’ concerns and sympathized with their challenges. - Open and ongoing communication, understanding, and CHWs who act as the direct link between the project and the community has been fundamental in balancing community needs and requests with the scope and aims of a research project |
| 7 | Marsh et al. 2011 | - Ethical dilemmas are continuously generated as part of community engagement activities, including the risks of perverse outcomes related to existing social relations in communities and conditions of ‘half knowing’ intrinsic to processes of developing new understandings | - Community engagement is an important process to address this issue and supports the enactment of normative roles, but it requires a broad approach to build mutual understanding and trust between researchers and community members |
| 8 | Meiring et al. 2019 | - Despite community engagement activities and community leaders sharing information and answering questions, there were still misconceptions within the community | - Providing information and answering questions at enrolment was important for addressing misconceptions and helping individuals to make informed decisions. Information provided at enrollment travels through the wider community as those messages were delivered back to friends and family by participants |
| 9 | Molyneux et al. 2016 | - Identifying and working with different MSM sub-groups risks inappropriate labeling and the creation of false categories that do not resonate with reality or that benefit some at the expense of others, and possibly raising stigma and discrimination - There are a range of ethical challenges in engaging with these diverse communities | - Honest and open communication about what the goals of engagement are and what can be achieved for MSM communities through research, given what are often critical and multi-faceted needs; - Ensuring that what is promised and given to MSM participants and communities as part of research is not organized in such a way as to undermine individuals’ abilities to make free and informed choices about whether or not to participate in that research; - Avoiding being drawn into internal conflicts within and between different LGBT/GMT groups in such a way as to undermine research benefits or increase the vulnerability of MSM participants or communities; and - Seeking out and taking into account the views and priorities of arguably the most vulnerable MSM - Explaining to the general population the public health reasoning for working with MSM can be misinterpreted as the promotion of homosexuality which is highly sensitive in many homophobic contexts - It is essential that key messages to these groups are carefully worded to minimize negative stereotyping and misconceptions that reinforce stigma |
| 10 | Mtove et al. 2018 | Some of the challenges in multinational research include:   - Challenges related to coordination with ongoing global malaria efforts, heterogeneity in national regulatory structures, sub-optimal healthcare infrastructure, local practices and beliefs, and perspectives that view healthcare providers with undue trust or suspicion. - A major challenge faced in running a clinical trial in resource-limited settings is navigating language and literacy issues in obtaining informed consent - Another challenge was the follow-up. Locating the participants’ residences was a challenge given that in most of our research areas, residences lack clear directions, street names, or house numbers | - Implementation of a range of engagement measures to ensure compliance with global clinical and regulatory standards - Participants consented to using their language of choice after confirming an acceptable literacy level - In both urban and rural locations, field workers had to escort the participants to their homes and make a map that they could follow for the next dosing days and home check-ups respecting the participants and their families’ choices |
| 11 | Nakibinge et al. 2009 | - The community’s tendency to suffer from participation fatigue increases with time | - Counter-measures to maintain interest and coverage rates such as introducing new incentives (e.g., mosquito nets); improving the quality of services provided to the community; and developing new research ideas. - Ongoing engagement enables the identification of those project components that promote continued participation - The field staff’s tendency to suffer from participation fatigue is countered by annual assessments of staff training needs and investment in capacity development |
| 12 | Nyika et al. 2010 | - In community engagement there could be potential challenges such as lengthy processes and expectations that may be too high to meet | - Community engagement by itself is an appropriate platform to address such challenges |
| 13 | Okello et al. 2013 | - Both practical and ethical challenges were encountered during consenting and assenting procedures | - Strategy for addressing the challenges focused on improving communication and understanding of the trial, and maintaining dialogue with all the relevant stakeholders throughout the study period |
| 14 | Olaseha and Sridhar 2005 | - There was lack of funding to implement planned activities; lack of continuity of programs; community needs are not always in line with Funding Agencies’ agenda; and the Community Based Organizations (CBOs) are interested more in services than research, while the academics are interested more in research than services; and a sustainable partnership is hard to build based on the above-mentioned challenges. | - From Faculty’s perspective, interests and activities of academics are expanded beyond campus environment, and this offers the opportunity for research and integration of theory and practice. |
| 15 | Pare et al. 2021 | - Some gaps in the analysis of power dynamics and relationships between different social levels and how this could impact the decision-making processes. | - Informal assessment of   the information understanding was carried out, based on feedbacks from stakeholders and conversations between engagement team and community members |
| 16 | Shahmanesh et al. 2021 | - *Thetha Nami* ‘*talk to me’* not being valued by youth, seen as “useless by youth” | - The peer – Navigators deconstructed what these challenges signified. They identified this as partly due to the logistic barriers they faced in delivering Thetha Nami: small age differences particularly with older adolescents; lack of safe spaces for youth to gather; and size of the rural areas with poor transport links. - On reflection; they identified the “uselessness” as signifying gaps in the services they had at their disposal to offer youth. They fill those gaps. |
| 17 | Simwinga et al. 2016 | - In Zambia, Civil Society Organizations (CSO) were concerned about the possible negative effects of early treatment in people living with HIV and advocated for their involvement in monitoring - CAB members from the control arm complained that   they were not well informed about what was happening in the study and felt the approach was disrespectful   - Rumors related to blood drawn from research participants were circulating in both countries in the first study year, including concerns that the blood was being used for satanic rituals or to stock up hospital blood banks | - A separate advisory structure, called the Community Partners Platform (CPP), was created to bring the civil society perspective into the consultations about study implementation, with a secretariat hosted by the Treatment Advocacy and Literacy Campaign (TALC) - Additional field staff were employed to facilitate engagement in the Control Arm. CAB meetings also became more frequent-monthly instead of quarterly. Study sensitization activities were introduced with care taken to ensure that messaging was relevant to control community activities. - CAB members visited the study laboratories and were taken through the receiving, processing, and storage procedures. |
| 18 | Yotebieng et al. 2019 | - The outcomes of the process are strongly shaped by those who are most engaged - Sustaining participation across rounds is a known challenge | - Multi-disciplinary perspectives of an extended network of implementation experts, researchers, policy/decision makers, Advocates, and other stakeholders were engaged. - The process also facilitated the participation of researchers from both English and French-speaking contexts, with 20% of those participating in the final round being French-speakers. |
