# Supplementary material for: Community engagement in research addressing infectious diseases of poverty in sub-Saharan Africa: A qualitative systematic review
Source: PLOS Glob Public Health. 2024 Jul 15;4(7):e0003167. doi: 10.1371/journal.pgph.0003167 (PMC11249264; doi:10.1371/journal.pgph.0003167)
Supplement: S1 Text — (DOCX) [file pgph.0003167.s008.docx]

**S1 Text:** Excluded Studies with the reason for exclusion

Bardosh KL, Scoones JC, Grace D, Kalema-Zikusoka G, Jones KE, de Balogh K, et al. Engaging research with policy and action: what are the challenges of responding to zoonotic disease in Africa? Philosophical Transactions of the Royal Society B: Biological Sciences. 2017; 372 (1725):20160172.

***Reason for exclusion****: Ineligible phenomena of interest*

Bedwell C, Lavender T. Giving patients a voice: implementing patient and public involvement to strengthen research in sub-Saharan Africa. Journal of Epidemiology and Community Health. 2020; 74(4):307–10.

***Reason for exclusion****: Ineligible study design (Commentary) and outcome*

Breuer A, Asiedu E. Can Gender-Targeted Employment Interventions Help Enhance Community Participation? Evidence from Urban Togo. World Development. 2017; 96:390–407.

***Reason for exclusion****: Ineligible phenomena of interest and outcome*

Esienumoh EE, Allotey J, Waterman H. Empowering members of a rural southern community in Nigeria to plan to take action to prevent maternal mortality: A participatory action research project. Journal of Clinical Nursing. 2018; 27(7–8):E1600–11.

***Reason for exclusion****: Ineligible outcomes*

Folayan MO, Brown B, Haire B, Babalola CP, Ndembi N. Considerations for stakeholder engagement and COVID-19 related clinical trials’ conduct in sub-Saharan Africa. Developing World Bioethics. 2021; 21 (1):44–50.

***Reason for exclusion****: Ineligible study design (literature review)*

Folayan MO, Oyedeji KS, Fatusi OA. Community Members’ Engagement with and Involvement in Genomic Research: Lessons to Learn from the Field. Developing World Bioethics. 2015; 15 (1):1–7.

***Reason for exclusion****: Ineligible study design (literature review)*

Gooding K, Phiri M, Peterson I, Parker M, Desmond N. Six dimensions of research trial acceptability: how much, what, when, in what circumstances, to whom and why? Social Science & Medicine. 2018; 213:190–8.

***Reason for exclusion****: Ineligible phenomena of interest*

Groves AK, Hallfors DD, Iritani BJ, Rennie S, Odongo FS, Kwaro D, et al. “I think the parent should be there because no one was born alone”: Kenyan adolescents’ perspectives on parental involvement in HIV research. African Journal of AIDS Research [Internet]. 2018;17 (3):227–39. Available from: <https://doi.org/10.2989/16085906.2018.1504805>

***Reason for exclusion****: Ineligible phenomena of interest*

Hannan R. Participatory cooperative research: for the people, by the people, with the people. Development in Practice. 2015; 25(4):490–502.

***Reason for exclusion****: Ineligible outcomes*

Ismail MM, Gerrish K, Naisby A, Salway S, Chowbey P. Engaging minorities in researching sensitive health topics by using a participatory approach. Nurse Res. 2014; 22 (2):44–8.

***Reason for exclusion****: Ineligible context*

Kamuya DM, Molyneux CS, Sally T. Gendered negotiations for research participation in community-based studies: implications for health research policy and practice. BMJ Global Health. 2017; 2 (2).

***Reason for exclusion****: Ineligible phenomena of interest*

Lairumbi GM, Molyneux S, Snow RW, Marsh K, Peshu N, English M. Promoting the social value of research in Kenya: examining the practical aspects of collaborative partnerships using an ethical framework. Soc Sci Med. 2008; 67(5):734–47.

***Reason for exclusion****: Ineligible phenomena of interest*

McCall B. Profile: MLW optimises community engagement in research. Lancet. 2012; 380(9839):328.

***Reason for exclusion****: Ineligible study design (Commentary) and outcome*

Mosavel M, Simon C, van Stade D, Buchbinder M. Community-based participatory research (CBPR) in South Africa: engaging multiple constituents to shape the research question. Soc Sci Med. 2005; 61(12):2577–87.

***Reason for exclusion****: Ineligible outcomes*

Mulenga P, Boelaert M, Lutumba P, Vander Kelen C, Coppieters Y, Chenge F, et al. Integration of Human African Trypanosomiasis Control Activities into Primary Health Services in the Democratic Republic of the Congo: A Qualitative Study of Stakeholder Perceptions. Am J Trop Med Hyg. 2019; 100(4):899–906.

***Reason for exclusion****: Ineligible phenomena of interest*

Munung NS, Chi PC, Abayomi A, Afolabi MO, Ambe J, Bonarwolo K, et al. Perspectives of different stakeholders on data use and management in public health emergencies in sub-Saharan Africa: a meeting report. Wellcome Open Research. 2021; 6:11–11.

***Reason for exclusion****: Ineligible phenomena of interest*

Nostlinger C, Loos J. Involving lay community researchers in epidemiological research: experiences from a seroprevalence study among sub-Saharan African migrants. AIDS Care-Psychological and Socio-medical aspects of AIDS/HIV. 2016; 28:119–23.

***Reason for exclusion****: Ineligible context*

Nyblade L, Singh S, Ashburn K, Brady L, Olenja J. “Once I begin to participate, people will run away from me”: Understanding stigma as a barrier to HIV vaccine research participation in Kenya. VACCINE. 2011;29(48):8924–8.

***Reason for exclusion****: Ineligible phenomena of interest*

Ogunrin O, Woolfall K, Gabbay M, Frith L. Relative solidarity: Conceptualizing communal participation in genomic research among potential research participants in a developing Sub-Saharan African setting. PLOS ONE. 2018;13(4).

***Reason for exclusion****: Ineligible phenomena of interest and outcome*

Okell CN, Pinchbeck GP, Stringer AP, Tefera G, Christley RM. A community-based participatory study investigating the epidemiology and effects of rabies to livestock owners in rural Ethiopia. Prev Vet Med. 2013; 108(1):1–9.

***Reason for exclusion****: Ineligible phenomena of interest*

Oliphant SM, Donaldson LP. A Community-Based Participatory Approach to Understanding HIV/AIDS in the Ethiopian Community. Soc Work Public Health. 2019; 34(7):557–69.

***Reason for exclusion****: Ineligible context*

Omale UI, Azuogu BN, Alo C, Madubueze UC, Oka OU, Okeke KC, et al. Social group and health-care provider interventions to increase the demand for malaria rapid diagnostic tests among community members in Ebonyi state, Nigeria: a cluster-randomised controlled trial. Lancet Glob Health. 2021; 9(3):e320–30.

***Reason for exclusion****: Ineligible phenomena of interest*

Patterson AS. Engaging therapeutic citizenship and clientship: Untangling the reasons for therapeutic pacifism among people living with HIV in urban Zambia. Glob Public Health. 2016; 11(9):1121–34.

***Reason for exclusion****: Ineligible phenomena of interest and outcome*

Smit J, Middelkoop K, Myer L, Seedat S, Bekker L, Stein J. Willingness to participate in HIV vaccine research in a peri-urban South African community. International Journal of STD & AIDS 2006; 17: 176–179.

***Reason for exclusion****: Ineligible phenomena of interest and outcome*

Slack C, Strode A, Grant C, Milford C. Implications of the ethical-legal framework for adolescent HIV vaccine trials--report of a consultative forum. S Afr Med J. 2005; 95(9):682–4.

***Reason for exclusion****: Ineligible phenomena of interest*

Thomas R, Skovdal M, Galizzi MM, Schaefer R, Moorhouse L, Nyamukapa C, et al. Improving risk perception and uptake of pre-exposure prophylaxis (PrEP) through interactive feedback-based counselling with and without community engagement in young women in Manicaland, East Zimbabwe: study protocol for a pilot randomized trial. Trials. 2019; 20(1).

***Reason for exclusion****: Ineligible study design (Protocol)*

Vanderslott S, Van Ryneveld M, Marchant M, Lees S, Nolna SK, Marsh V. How can community engagement in health research be strengthened for infectious disease outbreaks in Sub-Saharan Africa? A scoping review of the literature. BMC Public Health. 2021; 21(1).

***Reason for exclusion****: Ineligible study design (Scoping review)*

Vreeman R, Kamaara E, Kamanda A, Ayuku D, Nyandiko W, Atwoli L, et al.Community Perspectives on Research Consent Involving Vulnerable Children in Western Kenya. Empir Res Hum Res Ethics. 2012; 7(4): 44–55.

***Reason for exclusion****: Author manuscript found to be a duplicate of another included study*

*Walzl* G, Beyers N, van Helden P. TB: a partnership for the benefit of research and community. Trans R Soc Trop Med Hyg. 2005;99 Suppl 1:S15-9.

***Reason for exclusion****: Ineligible phenomena of interest*
