# Supplementary material for: Community engagement in research addressing infectious diseases of poverty in sub-Saharan Africa: A qualitative systematic review
Source: PLOS Glob Public Health. 2024 Jul 15;4(7):e0003167. doi: 10.1371/journal.pgph.0003167 (PMC11249264; doi:10.1371/journal.pgph.0003167)
Supplement: Attachment — Submitted filename: Community Engagement.docx [file pgph.0003167.s009.docx]

**Title**

The title "Community Engagement in Research Addressing Infectious Diseases of Poverty in Sub-Saharan Africa: A Qualitative Systematic Review" accurately reflects the content discussed in the sections the authors have provided It is clear that the review focuses on community engagement in research related to infectious diseases of poverty in the Sub-Saharan Africa region, with a particular emphasis on qualitative research methods.

**Abstract**

The abstract is well written and provides a clear description of the study. However, there are some areas that could be improved:

1. 1. Context and Rationale: While the executive summary notes the importance of community participation in research and the lack thereof in sub-Saharan Africa, it could provide more context as to why this is an important issue. For example, you could briefly discuss the impact of infectious diseases on poverty in sub-Saharan Africa and how community engagement could potentially address these issues.
2. Methodology: In the abstract, the authors could provide more details about the methodology. For example, they could mention the specific databases they searched, and the criteria used to select the studies. This would give readers a better understanding of the scope and accuracy of the review.
3. Findings: The abstract could present the findings in a more structured manner. You could categorize the results according to different phases of the research (e.g., conceptualization, analysis, dissemination, interpretation) and discuss the level of community involvement at each phase.
4. Implications: The abstract could discuss the implications of the findings in more depth. It could talk about how the findings could inform future research efforts and policymaking in SSA.
5. Recommendations: The abstract ends with a call for more primary studies on community engagement. It could also suggest specific areas for future research based on the gaps identified in the review.

**Introduction**

here are some of my comments and suggestions based on the introduction:

-The introduction provides a good background and rationale for the review, but it could be more focused and coherent. You could start by defining the key terms and concepts of community engagement, infectious diseases of poverty, and Sub-Saharan Africa, and explain why they are important and relevant for your review. Then, you could present the existing evidence and gaps on community engagement in research addressing infectious diseases of poverty in Sub-Saharan Africa, and how your review aims to fill those gaps and contribute to the literature. Finally, you could state your research questions and objectives more clearly and explicitly and provide an outline of the rest of the paper.

- You can also improve the readability and flow of your introduction by using transitions and connectors between paragraphs and sentences and avoiding repetition and redundancy. For example, you could use words like “however,” “therefore,” “so,” “additionally,” “moreover,” etc. to connect your ideas and arguments. You can also avoid repeating the same expressions or sentences, such as: E.g., “Community Participation in Research,” “Infectious Diseases of Poverty,” “Low- and Middle-Income Countries,” etc. using synonyms or pronouns.

- You could also use some headings to organize the introduction into sections, such as Background, Literature Review, Research Gap, Research Questions and Objectives, and Paper Outline. This would make it easier for the readers to follow the structure and logic of your review.

- Line 98-124- The paragraphs provide some background information on the challenges and gaps of community engagement in research addressing infectious diseases of poverty in LMICs and SSA. However, it could be more concise and clearer by avoiding repetition and redundancy. For example, you could merge the sentences Lines 98-100 and Lines 102-103 into one sentence that summarizes the main problem of poor community engagement in research.

- The paragraphs could also use some transitions and connectors to link the ideas and arguments more smoothly. For example, you could use words like "however", "therefore", "thus", "in addition", "moreover", etc. to show the relationship between the sentences and paragraphs. You could also use signposting words to indicate the structure and logic of your review, such as "firstly", "secondly", "finally", etc.

- The paragraphs could also state the research questions and objectives more clearly and explicitly and provide an outline of the rest of the paper. This would help readers understand the purpose and scope of your review and what to expect from the following sections.

Here is a possible revision of the paragraphs based on my comments and suggestions:

*However, despite the potential benefits of community engagement in research, there is a lack of effective and sustained engagement of communities in research addressing infectious diseases of poverty, especially in LMICs and SSA. This leads to poor alignment of research with community needs and priorities, low uptake of research evidence for decision-making, and limited health improvements and capacity building of individuals and institutions [19,24–33]. Furthermore, there is a lack of empirical evidence on how to engage communities in research in these settings and what barriers and facilitators hinder community engagement in research [35]. Therefore, this review aims to summarize existing evidence on experiences and practices in community engagement in infectious diseases of poverty research in sub-Saharan Africa and to identify challenges and lessons learned from engaging communities in health research. To achieve this goal, this paper addresses the following research questions:*

*1) What are the community engagement activities and experiences in research for addressing infectious diseases of poverty in SSA?*

*2) What are the challenges encountered and the mitigation strategies applied in community-engaged research addressing infectious diseases of poverty in SSA?*

**Methods**

Overall, the methodology section is well written and provides a detailed description of how the systematic review was conducted. Adheres to recognized standards and operates transparently. It effectively communicates the steps taken to ensure rigor and reliability in the review process. The methodology section you provided is comprehensive and covers all the essential aspects of conducting a systematic review.

However, there are a few minor suggestions that could enhance clarity and completeness:

1. Justification for the JBI Methodology: It might be beneficial to briefly explain why the JBI (Joanna Briggs Institute) methodology was chosen for this review. This could help provide context for readers who may be unfamiliar with it.
2. Rationale for PICo Framework: While you mention that the eligibility criteria are based on the PICo framework, you could briefly explain why this framework was chosen and how it is particularly relevant to the objectives of this review.
3. Publication Date Rationale: You mention that studies were limited to those published or conducted since January 2005. Providing a brief rationale for this timeframe could help readers understand the choice.
4. Rationale for Language Restriction: Mentioning why studies published in English were specifically included could be beneficial. This could relate to accessibility for the reviewers or the anticipated primary audience.
5. Discussion of Potential Biases: While it's briefly mentioned that disagreements between reviewers were resolved, you might want to consider adding a sentence about how potential biases or conflicts of interest were addressed in the study selection and assessment process.
6. Consideration of Grey Literature: Since grey literature searches were conducted, you could briefly explain the importance of including non-peer-reviewed sources and how this contributes to the comprehensiveness of the review.

Remember, these suggestions are relatively minor, and the methodology section is already well-structured and thorough. Incorporating these points would enhance the completeness and clarity of the section, but they are not critical omissions.

**Results.**

The authors have provided a detailed account of the community engagement activities in the research. This level of detail is great for understanding the depth of community involvement in the studies. The examples and specific actions taken to engage the community are very informative.

However, given the extensive nature of this section, it might be beneficial to consider the following:

1. Subheading Clarity: Ensure that the subheadings are clear and distinct. This helps the reader easily navigate through the section.
2. Formatting for Readability: You may want to consider breaking down the information into smaller paragraphs for better readability. This could help prevent information overload.
3. Consistency in Citation Style: Make sure that the citations for each study are consistent in style and format.
4. Explicit Mention of Study References: You're providing detailed information about various studies. It could be helpful to explicitly mention the reference (e.g., [Author(s), Year]) at the beginning of each study's description.
5. Incorporating Tables or Figures: Consider if there are any tables or figures that could be used to summarize or complement the information provided.

Remember, providing detailed descriptions is valuable, but maintaining clarity and readability is equally important. Additionally, consider whether this level of detail aligns with your overall goals and the expectations of your audience.

Ethical dilemmas and consenting.

In this section, you provide valuable insights into the ethical challenges encountered in engaging vulnerable populations in culturally sensitive research. To enhance readability and comprehension, consider breaking down some of the longer sentences (e.g., lines 848-852) for better clarity. Additionally, ensure that all sources are properly cited throughout the section for credibility (line 859).

Practical and ethical challenges during consenting and assenting procedures

Continue to highlight the practical and ethical challenges faced during consenting and assenting procedures. It would be beneficial to add brief reflections or analyses after presenting each challenge, indicating how it was addressed or its broader implications. Additionally, consider incorporating transitional phrases or sentences to improve the flow between different points (lines 860-872).

Navigating language and literacy issues

To address the challenge of navigating language and literacy issues in obtaining informed consent, emphasize the importance of consenting participants in their preferred language and confirming an acceptable literacy level (lines 873-876).

Ethical dilemmas and community engagement

Highlight the importance of community engagement in addressing ethical dilemmas. Emphasize the need for a broad approach to building mutual understanding and trust between researchers and community members (lines 878-882).

Communities' perceptions and knowledge of research

Continue to explore factors influencing a community's willingness to participate in research. Ensure that all cultural values are recognized and understood, especially across different age groups within a community (lines 883-895). Additionally, consider incorporating a brief reflection on how challenges were overcome, as shown in the example provided (lines 894-895).

Poor representativeness and impartiality of advisory board

Emphasize the importance of ensuring the representativeness and impartiality of advisory boards by involving locally sensitive recruitment and consultations with communities (lines 912-916). Consider adding a brief reflection on how power dynamics and relationships were assessed to prevent challenges (lines 916-920).

Lengthy research process and high community expectations

Continue to address challenges related to lengthy research processes and high community expectations. Highlight the importance of ongoing communication, understanding, and the role of Community Health Workers in balancing community needs with research scope (lines 921-932).

Participation fatigue

Elaborate on the strategies used to combat participation fatigue. Emphasize the importance of introducing incentives, improving service quality, and developing new research ideas to maintain community interest (lines 937-944).

For example, you could say *“To combat participation fatigue, researchers can employ several strategies to maintain community interest and motivation. This includes offering appropriate incentives, ensuring they align with community needs and are culturally sensitive. Additionally, providing high-quality services through effective communication, timely provision of services, and prompt issue resolution builds trust and strengthens the relationship between the research team and the community. Actively involving community members in the research design phase fosters a sense of ownership and investment. Introducing innovative research approaches and methodologies sparks curiosity and engagement. Regular communication, sharing progress updates, and seeking feedback demonstrate transparency and keep the community informed. Tailoring engagement strategies to diverse subgroups within the community acknowledges their unique needs and preferences. Moreover, continuously assessing the research's relevance to the community's priorities and needs ensures sustained interest. Implementing these strategies cultivates a positive, enduring partnership between researchers and the community, ultimately enhancing the quality and impact of the research endeavors.”*

Lack of shared control by the community

Highlight the significance of establishing trust with stakeholders and understanding the changing needs of the community to overcome challenges related to shared control (lines 948-952).

Lack of funds and shared priorities

Expand on the challenges posed by a lack of funds and differing priorities between communities and researchers. Stress the importance of broadening academic interests and activities beyond the campus environment (lines 953-960).

Late communication of study results

Provide further details on how the challenge of late communication of study results was addressed. Highlight the value of analyzing and publishing secondary data in audience-appropriate formats (lines 963-964).

These suggestions aim to enhance the clarity, coherence, and depth of your section on challenges in community engagement in research.

**Discussion**

Your discussion section provides a detailed and thorough analysis of community engagement in research addressing health care delivery in infectious diseases of poverty in SSA. It is well-structured and supported by references. Here are some specific suggestions for improvement:

1. Incorporate More Specific Examples: While you do provide examples throughout the discussion, consider incorporating more specific case studies or real-world examples to illustrate your points. This can help to make your discussion more tangible and relatable.
2. Expand on Challenges and Solutions: For each of the challenges mentioned (e.g., poor representativeness, partiality of advisory board), consider expanding on the potential solutions or strategies to address them. This would provide a more balanced view of the issues at hand.
3. Emphasize the Importance of Training: You mention the need for training and capacity development several times, which is crucial. You could further emphasize the significance of proper training and capacity building in ensuring effective community engagement.
4. Consider Formatting for Clarity: To enhance readability, consider breaking up longer sentences or paragraphs into smaller, digestible chunks. This can help readers absorb the information more easily.

For example, this is what I am referring to: [Line 1127 to 1140]

*Original Paragraph:*

*"In this review, we recognized that complete and comprehensive evidence and best practices with greater depth and breadth regarding community engagement in research is generally lacking; a limited spectrum of community engagement with low levels of participation, namely informing and consulting leaving communities without empowerment. Few studies achieved full-scale community engagement in the research process: some initiated the engagement at the design; most at implementations, and few at analysis and nearly nil in translations. Indeed, the review also identified significant barriers that potentially limited effective engagement, like, as poor representativeness and partiality of the advisory board; [14] absence of trust in the local health system; [12] lack of mutual understanding and trust between researchers and community members,[68,82] researchers inability and or lack of skills and knowledge regarding how to plan and implement participatory methods;[73] insufficient funds/research budgets dedicated to community engagement which could be due to researchers’ failure to properly cost community engagement activities in the research budget or poor attention by the funding agencies."*

*Revised Paragraph:*

*"In this review, we recognized that complete and comprehensive evidence and best practices with greater depth and breadth regarding community engagement in research is generally lacking. There's a limited spectrum of community engagement with low levels of participation, mainly focused on informing and consulting, which leaves communities without a sense of empowerment. Few studies achieved full-scale community engagement in the research process. Some initiated the engagement at the design phase, while most concentrated on implementation. There were very few instances of engagement at the analysis stage and nearly none in translations. Additionally, the review identified significant barriers that potentially limited effective engagement. These include issues such as poor representativeness and partiality of the advisory board,[14] absence of trust in the local health system,[12] and a lack of mutual understanding and trust between researchers and community members[68,82]. Researchers' inability or lack of skills and knowledge regarding how to plan and implement participatory methods[73] also posed a challenge. Moreover, insufficient funds allocated to community engagement may be attributed to researchers' oversight in properly budgeting for these activities, or it could result from inadequate attention by funding agencies."*

This revision breaks down the original paragraph into smaller, more focused sentences, making it easier for the reader to follow and absorb the information. It also helps to emphasize each point more effectively.

5. Highlight Implications for Future Research and Practice: Towards the end of the discussion, you could consider providing some reflections on the implications of your findings for future research and practice in the field of community engagement in health research in SSA.
6. Address Limitations Early: You mentioned limitations at the end. Consider discussing them earlier, perhaps after presenting your main findings. This way, readers are aware of potential constraints as they read through your analysis.

Remember, the discussion section is an opportunity to not only summarize your findings but also to critically analyze them and provide insights for the field.

**Conclusion**

This conclusion section provides a comprehensive summary of the key findings and offers practical implications for researchers and stakeholders involved in community-engaged research. It effectively highlights the importance of community engagement throughout the research process and acknowledges the existing challenges.

Here are a few suggestions to further enhance this section:

Sentence Clarity and Structure: Consider breaking some of the longer sentences into shorter ones to improve readability. For instance, in the second sentence, you could divide it into two for clearer emphasis on each point. Here's an example of how you can apply the suggestion to improve sentence clarity and structure:

*Original sentence: [Line 1180-1182]*

*"In precise narration, the communities and relevant stakeholders should be engaged in the initiation, conceptualization, designs of research methods and tools, implementation, dissemination, and translations of the findings."*

*Revised sentence:*

*"To provide a precise account, communities and relevant stakeholders should be engaged in various stages: initiation, conceptualization, design of research methods and tools, implementation, dissemination, and translation of findings."*

In this revised sentence, the information is broken down into shorter, more digestible segments, which can enhance readability and emphasize each stage of community engagement.

Specificity and Detail: Adding specific examples or case studies where applicable can help illustrate the points made in the conclusion. This can provide concrete evidence of successful community engagement practices.

Consistency and Repetition: There is some repetition of points across the conclusion section. While repetition can be effective for emphasis, make sure it doesn't become redundant.

An example of how you can apply the suggestion to address consistency and repetition:

*Original sentence:* *[Line 1179-1182]*

*"Effective and genuine community engagement in the research process should begin as early as possible. In precise narration, the communities and relevant stakeholders should be engaged in the initiation, conceptualization, designs of research methods and tools, implementation, dissemination, and translations of the findings."*

*Revised sentence:*

*"To ensure effective and genuine community engagement in the research process, it is crucial to initiate involvement as early as possible. Communities and relevant stakeholders should actively participate in the initiation, conceptualization, design of research methods and tools, implementation, dissemination, and translation of findings."*

In this revision, the repetition of the idea of community engagement beginning early in the research process is avoided, while still emphasizing the importance of this aspect. The information is presented in a more streamlined and coherent manner.

Implications for Future Research: Consider providing more specific suggestions for future research directions. For instance, what areas within community engagement could benefit from further exploration?

Here is an example of how you can provide more specific suggestions for future research directions within the context of community engagement:

*Original sentence: Line [1244-1247]*

*"Researchers may need to understand researchers' ability, understanding, skills, and attitude for community-engaged research. Likewise, future research needs to explore the community’s role in each stage of research with clear parameters; and effective strategies to reinforce research-related rules and regulations as engagement is a right of the community."*

*Revised sentence:*

*"For future research, it would be valuable to delve deeper into understanding researchers' proficiency, comprehension, skills, and attitudes necessary for effective community-engaged research. Additionally, there is a need to investigate the specific roles that communities can play in each stage of the research process, establishing clear parameters for their involvement. Furthermore, exploring effective strategies to reinforce research-related guidelines and regulations is imperative, as community engagement is not only a privilege but also a right of the community."*

In this revision, specific areas for future research are outlined, such as understanding researchers' abilities and exploring the community's roles in different stages of research. This provides a clearer and more focused direction for future studies on community engagement.

Clarification of Acronyms: Ensure that acronyms like "SSA" are defined or explained for readers who may not be familiar with them.

Engagement of Stakeholders: It might be beneficial to emphasize the importance of engaging not only community members but also other relevant stakeholders (e.g., policymakers, healthcare professionals) in the research process. Here's an example of how you can emphasize the importance of engaging various stakeholders in the research process:

*Original sentence: [Line 1237-1239]*

*"Engaging relevant and key stakeholders as early as possible at all levels of engagement and throughout the research process from inception to translation to policy or actions."*

*Revised sentence:*

*"In addition to involving community members, it is crucial to engage a wide range of stakeholders at every stage of the research process, starting from the project's inception to the translation of findings into actionable policies. This includes policymakers, healthcare professionals, advocacy groups, and other pertinent parties. By involving a diverse array of stakeholders, research efforts can be enriched with a broader perspective and ensure that outcomes are relevant and applicable to a wider audience."*

In this revision, the importance of engaging a variety of stakeholders, including policymakers and healthcare professionals, is highlighted. This provides a more comprehensive approach to community engagement in research.

Keep up the good work!

Good luck with your revisions!
